# Supplementary material for: FTO promotes weight gain via altering Kif1a splicing and axonal vesicle trafficking in AgRP neurons
Source: EMBO J. 2025 Jul 9;44(18):4919–61. doi: 10.1038/s44318-025-00503-3 (PMC12436618; doi:10.1038/s44318-025-00503-3)
Supplement: Supplementary file 1 — Table EV1 [file 44318_2025_503_MOESM1_ESM.pdf]

Table EV1

Sequences of the shHCR in situ hybridization probe for *Fto* and *Agrp*

Probe name	First probe	Second probe
Fto-1-S45	CCTCCACGTaaAGCCAAGTGTCTTCAAGCTCCTCAA	TCATCATCTTTGGGGGTCAGGTAAGaaTCCATCTAAGCT
Fto-2-S45	CCTCCACGTaaATTTCAGCTGCCACTGCTGATAGAA	CGGCCTCTCGGAAAACCAGTTTAGGaaTCCATCTAAGCT
Fto-3-S45	CCTCCACGTaaCCTTATGCAGCTCCTCTGGTATGCT	GCAGTGTGAGAAAGGCCTCGGGGACaaTCCATCTAAGCT
Fto-4-S45	CCTCCACGTaaTCCCGAAACAAGCAGCCATGCTTAT	ACATCTTTGCCTTGGATCCTCACCAaaTCCATCTAAGCT
Fto-5-S45	CCTCCACGTaaATGAGGATGCGAGACACTGGGGTGA	TACTTGTAGGTGCAGCCTGGGTCCCaaTCCATCTAAGCT
Fto-6-S45	CCTCCACGTaaGCACCGTGAAGAGTCTGGTGTTCAA	TGACCGTGCAGCCCTTCACGGGCCAaaTCCATCTAAGCT
Fto-7-S45	CCTCCACGTaaATGCAGCGGCGATCTCAGCCTCTGT	AGTCATTGAGCTTTAGGAAGGTCTGaaTCCATCTAAGCT
Fto-8-S45	CCTCCACGTaaTCTCTCTGACAGCCAGTTCTTCCAA	ACAGTGGCACAGCGTCTTCATTGGCaaTCCATCTAAGCT
Fto-9-S45	CCTCCACGTaaCGCCGGCCCTGGGGAACTCTGCCAT	CCACTTCATCATCGCAGGACGGCCCaaTCCATCTAAGCT
Fto-9-S45	CCTCCACGTaaGCAAAGTCACGTTGTAGGCTGCTCT	GCATCTTCTGAGGATCCATGAAGTTaaTCCATCTAAGCT
Fto-10-S45	CCTCCACGTaaCGAAATAGGGCTCCTCTTTCAAGTA	GCCAGCTCACCGCCATCTTCCCCATaaTCCATCTAAGCT
Fto-11-S45	CCTCCACGTaaGACCTGTCCACCAGGTTCTCATCGT	CAGCTATAGCTGTACACTGCCACGGaaTCCATCTAAGCT
Fto-12-S45	CCTCCACGTaaTCCTCACTTTCATCCTCAGAGCCTT	GGATCTCTGCCTTCGAAGCTGGACTaaTCCATCTAAGCT
Fto-13-S45	CCTCCACGTaaTTAAAACCAACATGCCAAGTATCAG	CCTGGTGTCTCGATGTCCCAAGAGAaaTCCATCTAAGCT
Fto-14-S45	CCTCCACGTaaTCCCTGGTGAAGAGGGATTGTTAAT	GAGGTCATCCAGCATGAAATAGCAGaaTCCATCTAAGCT
Fto-15-S45	CCTCCACGTaaCAAAACACAGTGCTGGTGGGTGGCA	GGAACTAAACCGAGGCTGTGAGCCAaaTCCATCTAAGCT
Fto-16-S45	CCTCCACGTaaGTTGAGCACTCTGCCACACGGTGAG	CGTTCTAAGATATAATCCAAGGTGCaaTCCATCTAAGCT
Fto-17-S45	CCTCCACGTaaGGACATTCTGCAGCGCCAACTGACA	AGACGTCGCCATCGTCTGAGTCATTaaTCCATCTAAGCT
Fto-18-S45	CCTCCACGTaaTTGTTTCAAAACTGCAGGATCAAAG	CTCCACCTCATTATGGATTTCCTCTaaTCCATCTAAGCT
Fto-19-S45	CCTCCACGTaaTGAAACCAGAACTGCCTCAGCCACT	TCGGTGCAAAGTTTGTATCGATTGCaaTCCATCTAAGCT
Fto-20-S45	CCTCCACGTaaAGGTGAGTCATGGGCTCACACCACC	CTCTCCATCTTCTTCCACAGCCCCTaaTCCATCTAAGCT
Fto-21-S45	CCTCCACGTaaCTTCACGGAGCACCGCATTTGTCAT	GTTCCACCGGGAGCCCCTCTCTTTTaaTCCATCTAAGCT
Fto-22-S45	CCTCCACGTaaATGGCAGACAGAATCTCACTCCTTT	TTCTGGCGCACGGTGAGCGGGACCAaaTCCATCTAAGCT
Agrp-1-S41	GCTCGACGTaaAACTCAGCAACATTGCAGTCAGCAT	TGGGAGGCAGTGCCAACAGCAGAACaaTCCTTTGCAACA
Agrp-2-S41	GCTCGACGTaaCCACGCCCATCTGGACCCCCAGTGT	CAGGCCTTCTGATGCCCTTCAGTGGaaTCCTTTGCAACA
Agrp-3-S41	GCTCGACGTaaGAACTCTGGGAACAGAGCCTGGTCA	CTTGAGGCCATTCAGACTTAGACCTaaTCCTTTGCAACA
Agrp-4-S41	GCTCGACGTaaCTGCTCGGTCTGCAGTTGTCTTCTT	CTTCTGCCTTCTGCAGCAGAACTTCaaTCCTTTGCAACA
Agrp-5-S41	GCTCGACGTaaGGATCTAGCACCTCCGCCAAAGCTT	CGCGGAGAACGAGACTCGCGGTTCTaaTCCTTTGCAACA
Agrp-6-S41	GCTCGACGTaaGGACTCGTGCAGCCTTACACAGCGA	GCAGCAAGGTACCTGCTGTCCCAAGaaTCCTTTGCAACA
Agrp-7-S41	GCTCGACGTaaCGGCAGTAGCACGTAGCGCACGGGT	CGGCAGTAGCAAAAGGCATTGAAGAaaTCCTTTGCAACA
Agrp-8-S41	GCTCGACGTaaCGTACCCAGCTTGCGGCAGTAGCAA	CTAGGTGCGACTACAGAGGTTCGTGaaTCCTTTGCAACA
Agrp-9-S41	GCTCGACGTaaCAGACTTAGACCTGGGAACTCTGGG	GTCTGCAGTTGTCTTCTTGAGGCCAaaTCCTTTGCAACA
Agrp-10-S41	GCTCGACGTaaACCTCCGCCAAAGCTTCTGCCTTCT	CGAGACTCGCGGTTCTGTGGATCTAaaTCCTTTGCAACA
