# Supplementary figures and images for: FTO promotes weight gain via altering Kif1a splicing and axonal vesicle trafficking in AgRP neurons
Source: EMBO J. 2025 Jul 9;44(18):4919–61. doi: 10.1038/s44318-025-00503-3 (PMC12436618; doi:10.1038/s44318-025-00503-3)

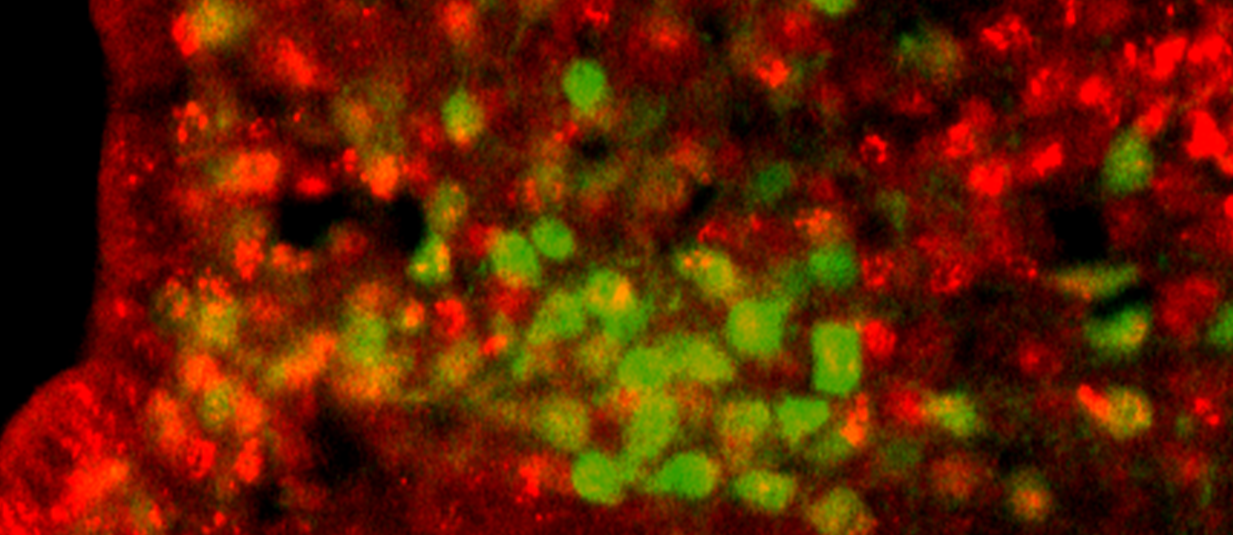

Supplement: Supplementary file 3 — Source data Fig. 1 [file 44318_2025_503_MOESM3_ESM.zip › Fig.1A-1.tif]

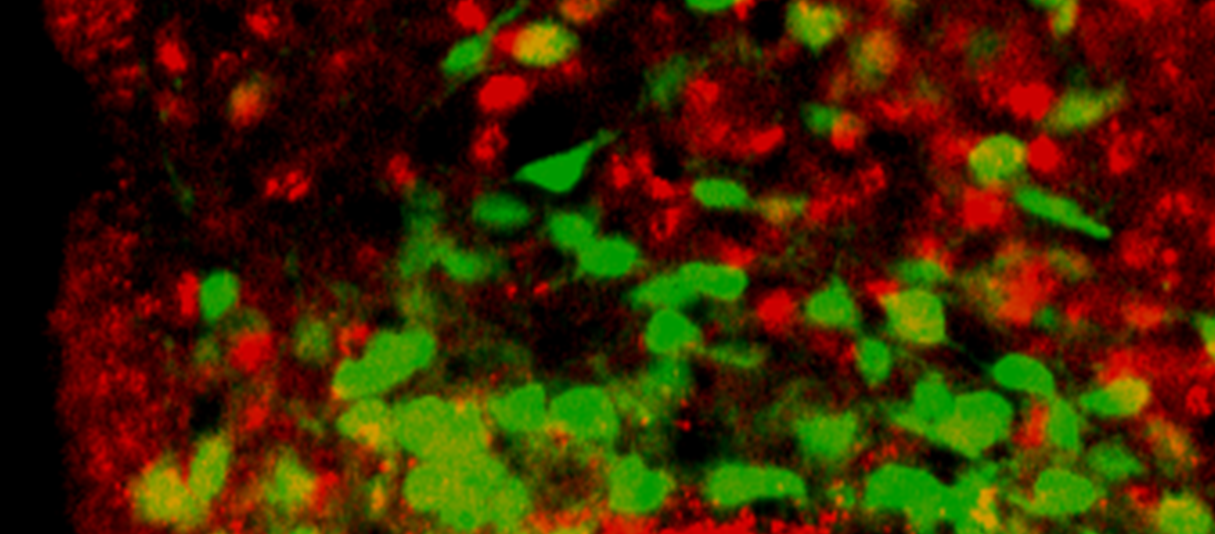

Supplement: Supplementary file 3 — Source data Fig. 1 [file 44318_2025_503_MOESM3_ESM.zip › Fig.1A-2.tif]

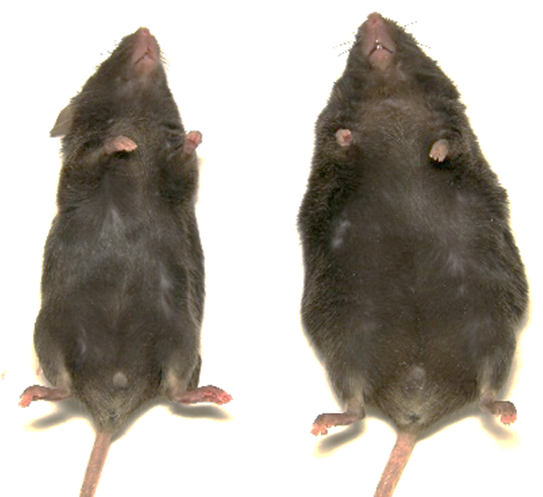

Supplement: Supplementary file 4 — Source data Fig. 2 [file 44318_2025_503_MOESM4_ESM.zip › Fig.2B.tif]

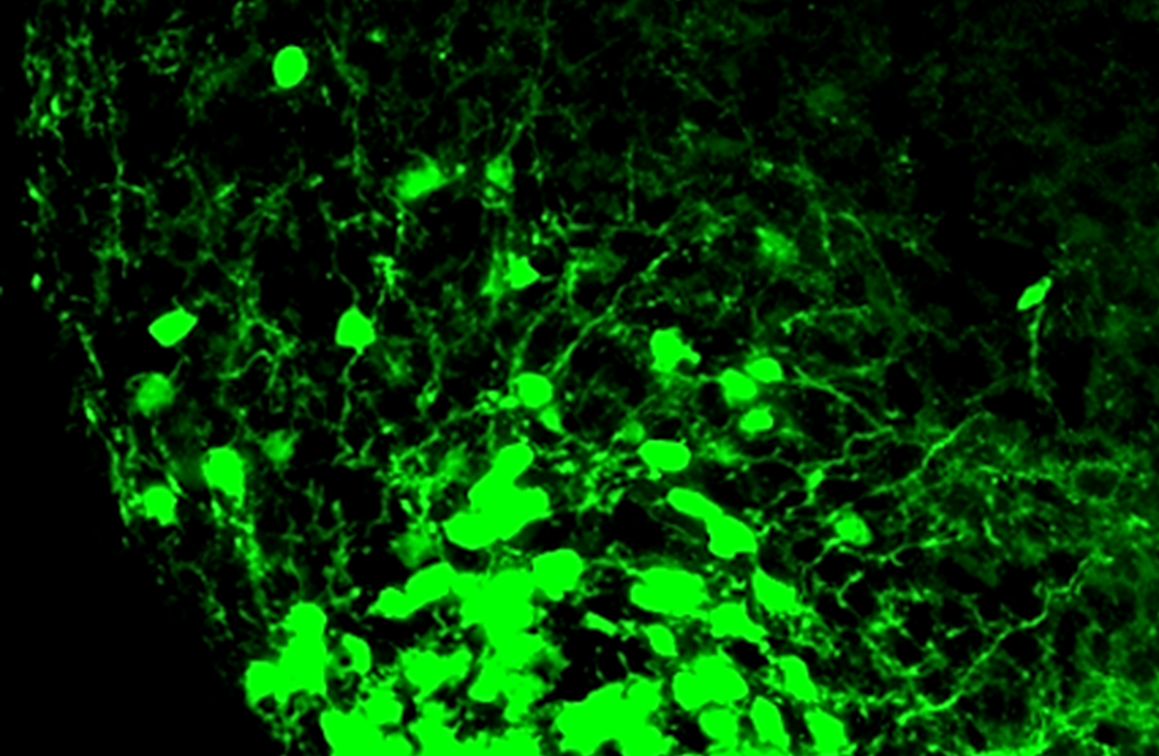

Supplement: Supplementary file 7 — Source data Fig. 5 [file 44318_2025_503_MOESM7_ESM.zip › Fig5A FtoloxloxAgrp-CreNPY-hrGFP.tif]

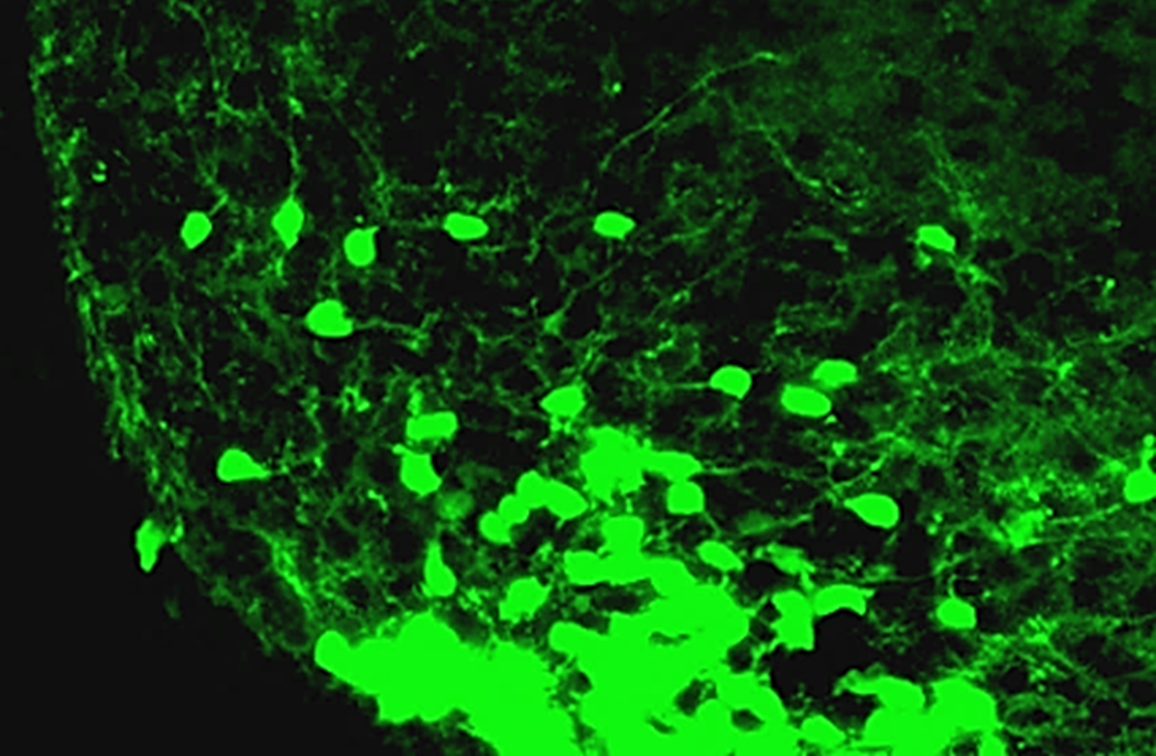

Supplement: Supplementary file 7 — Source data Fig. 5 [file 44318_2025_503_MOESM7_ESM.zip › Fig5A FtoloxloxNPY-hrGFP.tif]

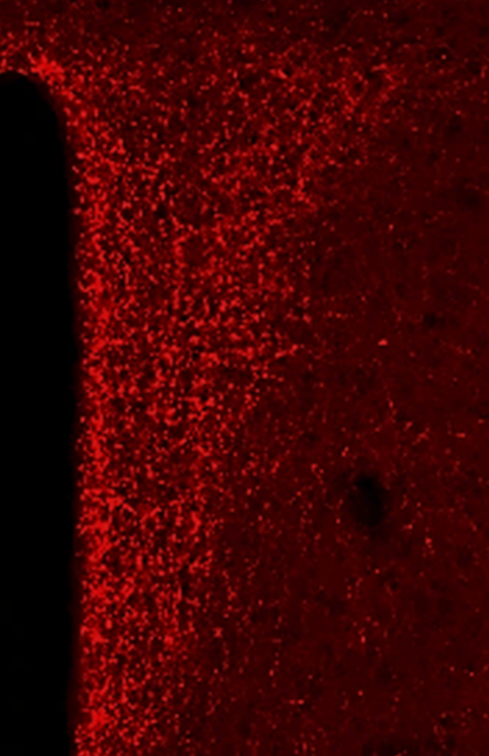

Supplement: Supplementary file 7 — Source data Fig. 5 [file 44318_2025_503_MOESM7_ESM.zip › Fig5D Ftoloxlox.tif]

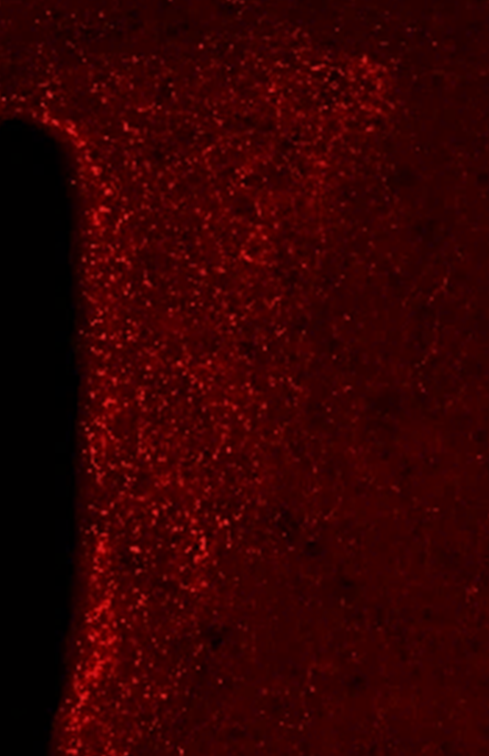

Supplement: Supplementary file 7 — Source data Fig. 5 [file 44318_2025_503_MOESM7_ESM.zip › Fig5D FtoloxloxAgrp-Cre.tif]

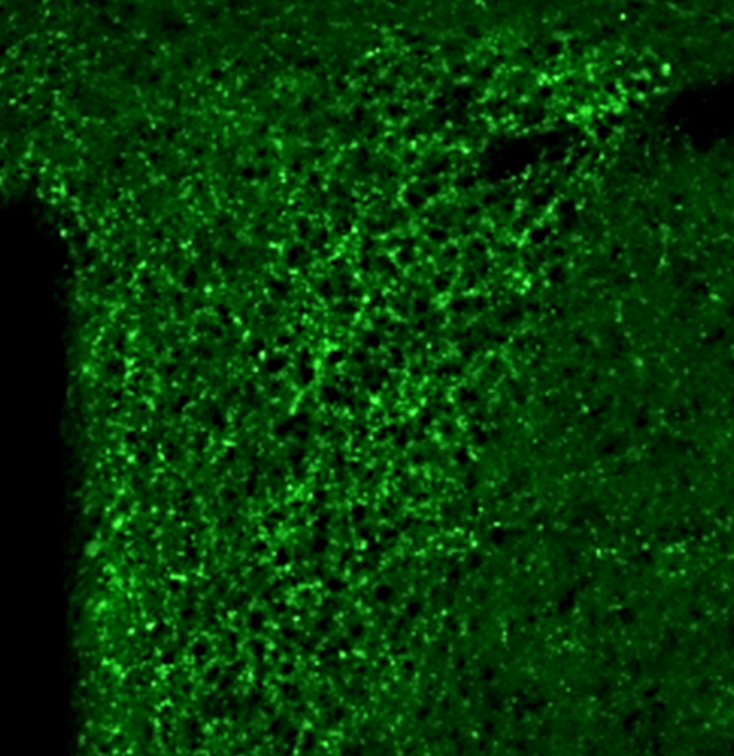

Supplement: Supplementary file 7 — Source data Fig. 5 [file 44318_2025_503_MOESM7_ESM.zip › Fig5F FtoloxloxAgrp-CreNPY-hrGFP NPY-hrGFP.tif]

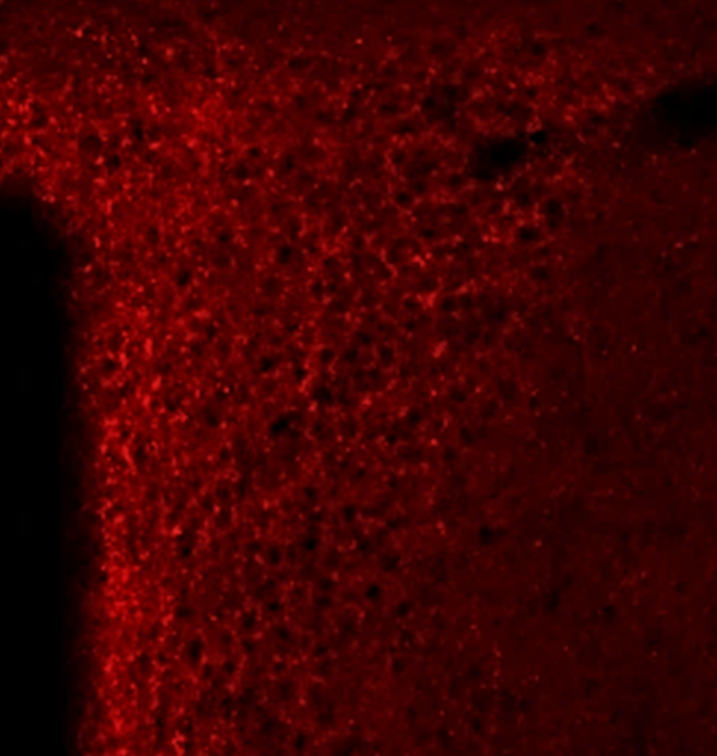

Supplement: Supplementary file 7 — Source data Fig. 5 [file 44318_2025_503_MOESM7_ESM.zip › Fig5F FtoloxloxAgrp-CreNPY-hrGFP Secretogranin II.tif]

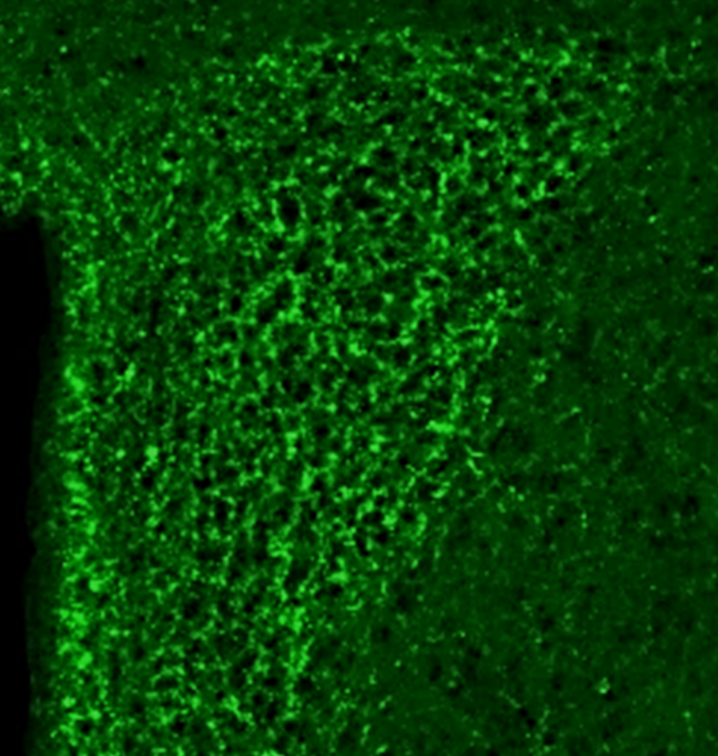

Supplement: Supplementary file 7 — Source data Fig. 5 [file 44318_2025_503_MOESM7_ESM.zip › Fig5F FtoloxloxNPY-hrGFP NPY-hrGFP.tif]

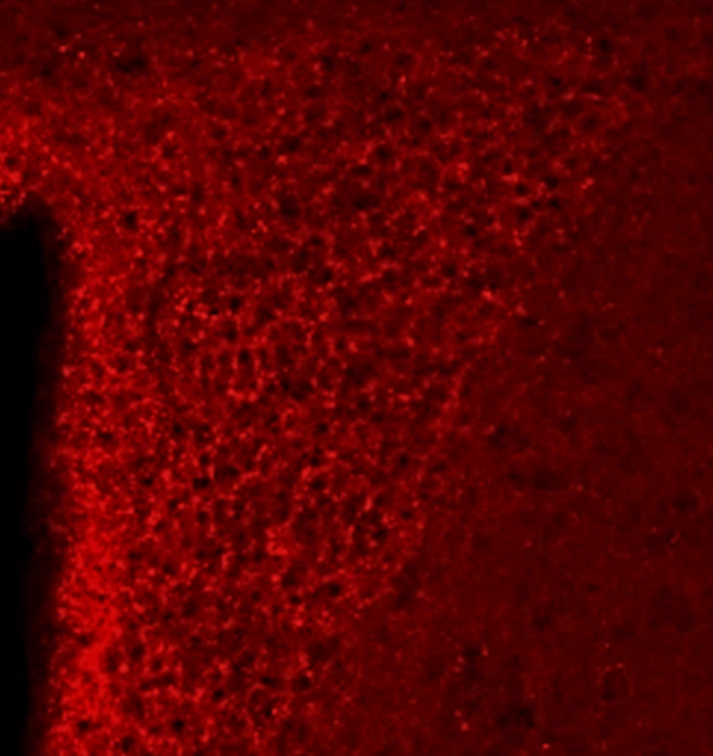

Supplement: Supplementary file 7 — Source data Fig. 5 [file 44318_2025_503_MOESM7_ESM.zip › Fig5F FtoloxloxNPY-hrGFP Secretogranin II.tif]

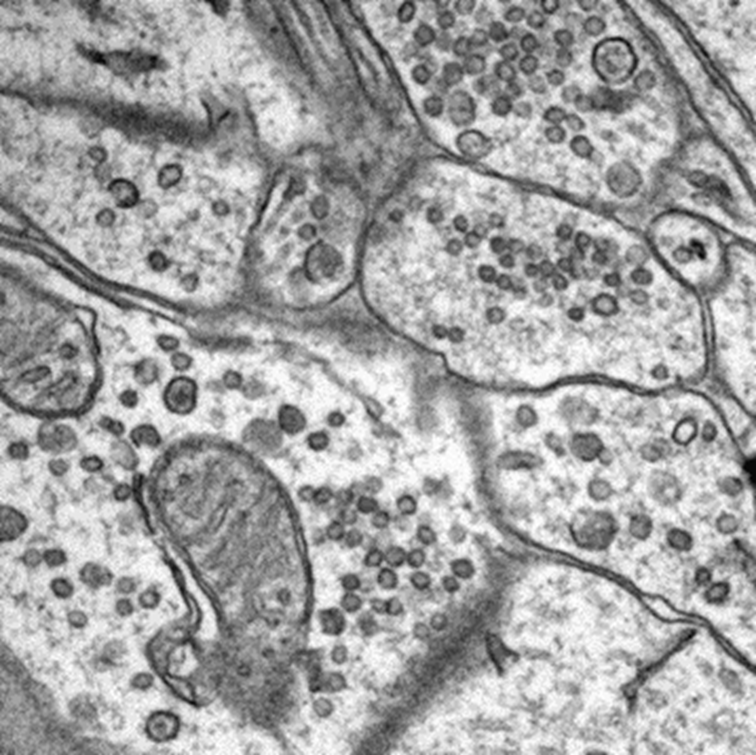

Supplement: Supplementary file 7 — Source data Fig. 5 [file 44318_2025_503_MOESM7_ESM.zip › Fig5H Ftoloxlox.tif]

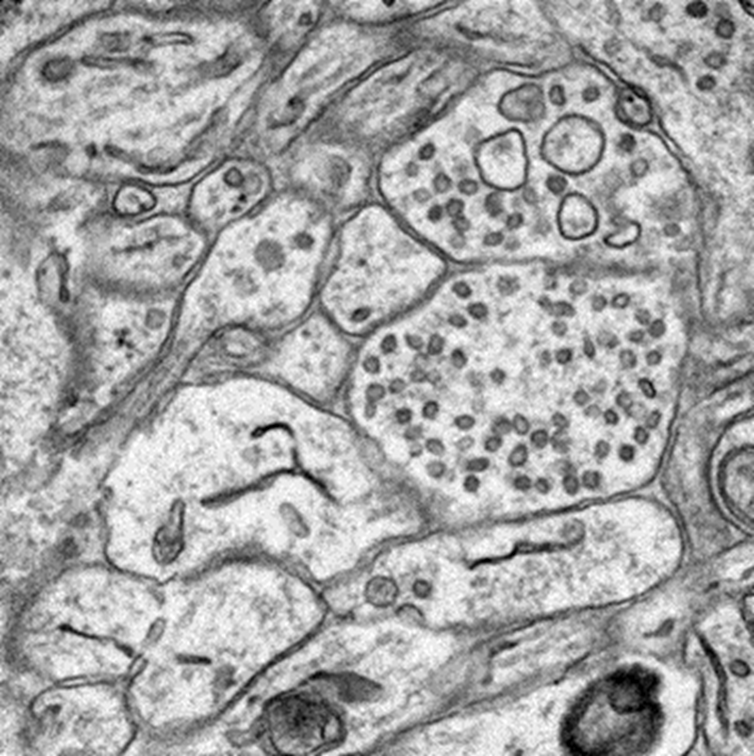

Supplement: Supplementary file 7 — Source data Fig. 5 [file 44318_2025_503_MOESM7_ESM.zip › Fig5H FtoloxloxAgrp-Cre.tif]

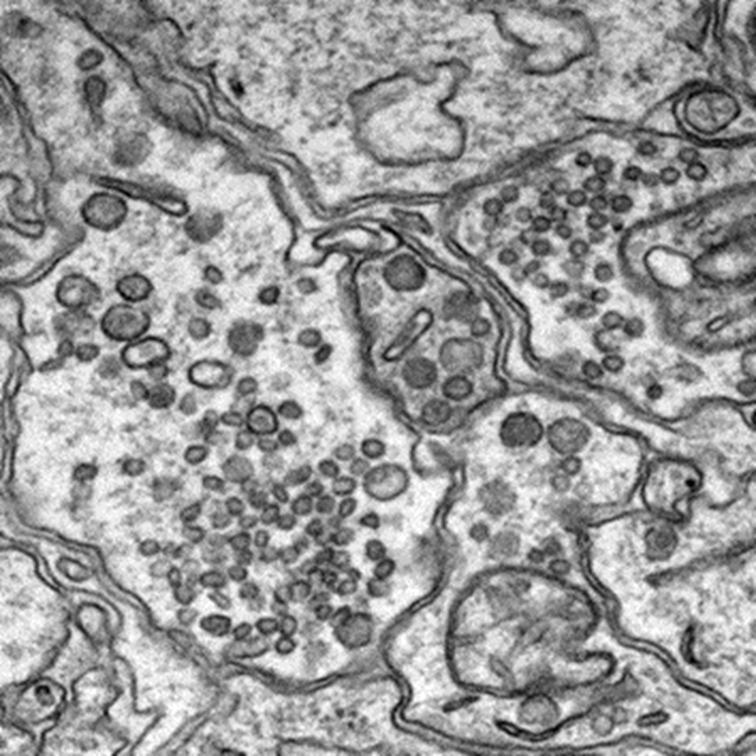

Supplement: Supplementary file 7 — Source data Fig. 5 [file 44318_2025_503_MOESM7_ESM.zip › Fig5J AAV-Flex-Fto-mCherry.tif]

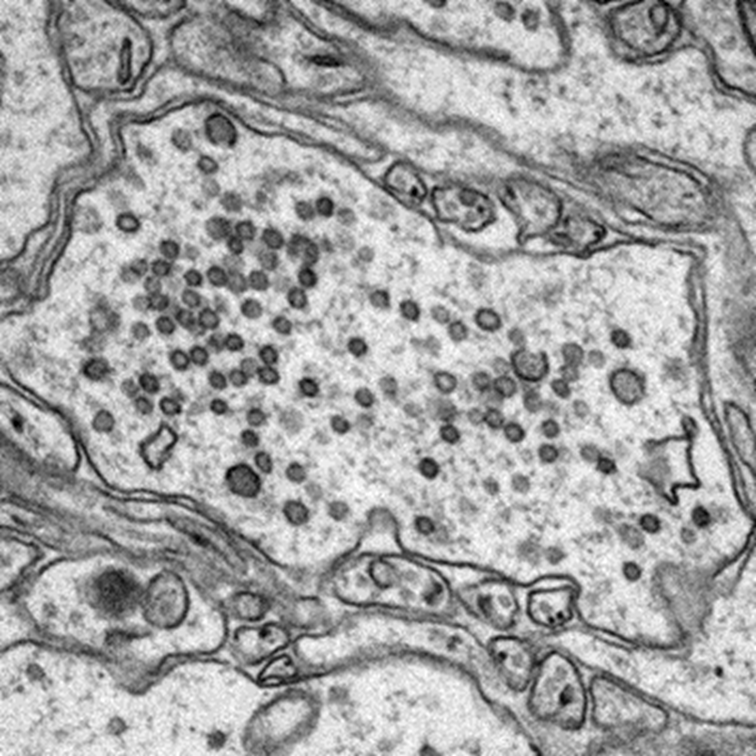

Supplement: Supplementary file 7 — Source data Fig. 5 [file 44318_2025_503_MOESM7_ESM.zip › Fig5J AAV-Flex-mCherry.tif]

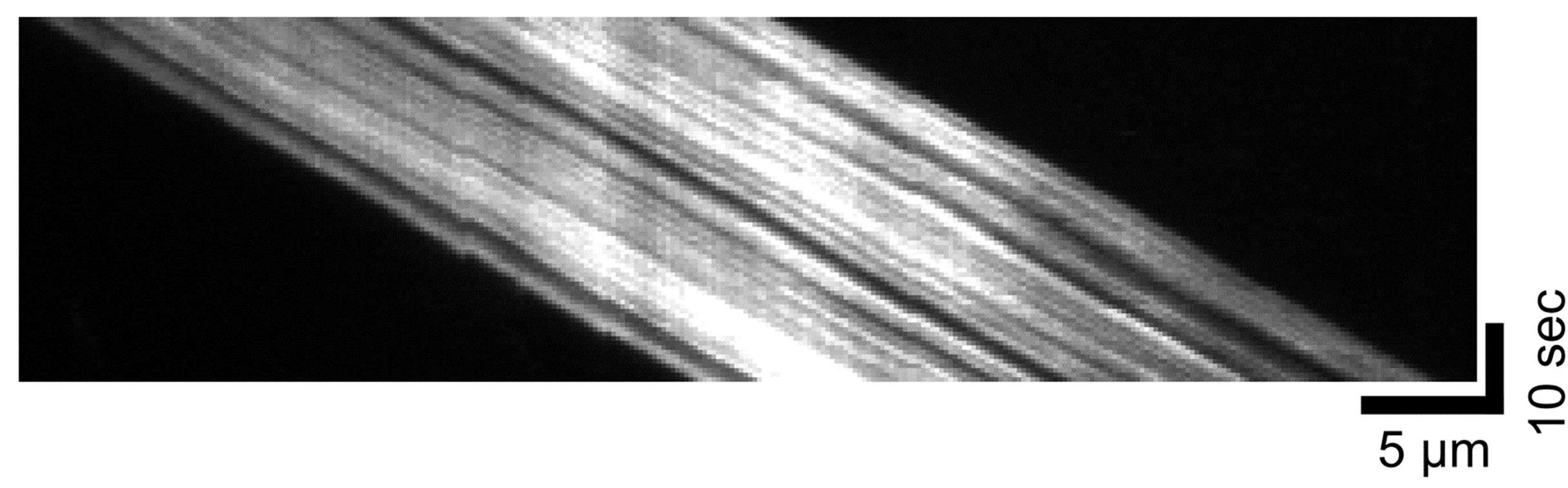

Supplement: Supplementary file 8 — Source data Fig. 7 [file 44318_2025_503_MOESM8_ESM.zip › Fig7E inclusion.tif]

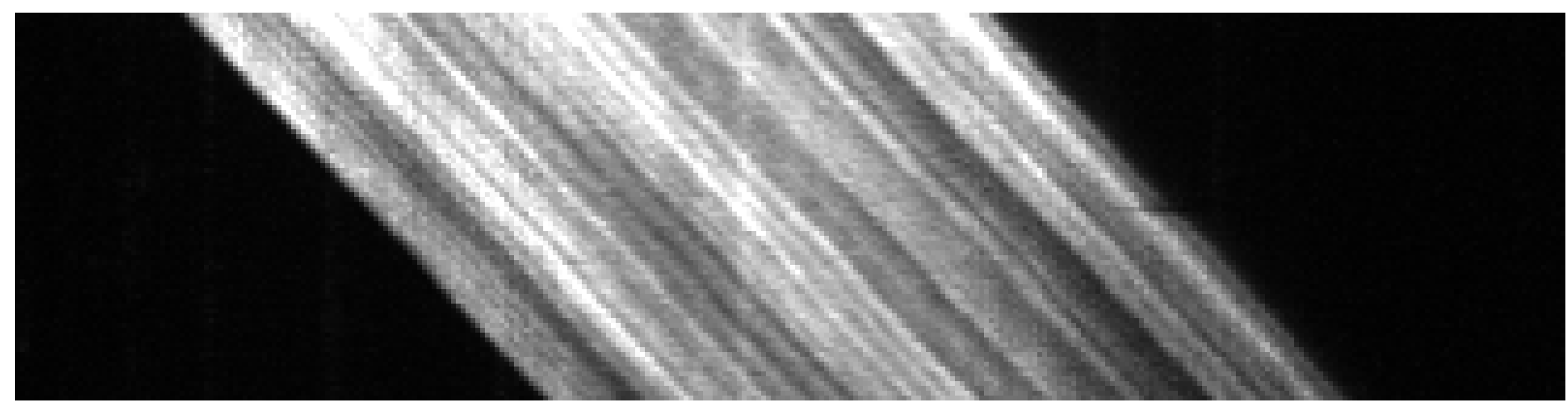

Supplement: Supplementary file 8 — Source data Fig. 7 [file 44318_2025_503_MOESM8_ESM.zip › Fig7E skipping.tif]

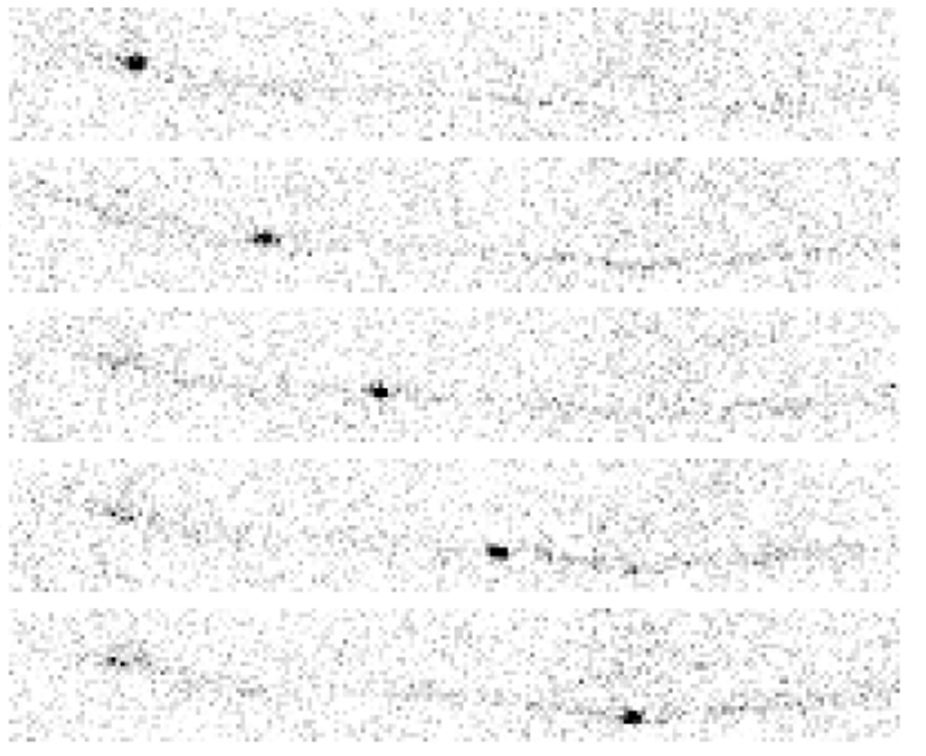

Supplement: Supplementary file 8 — Source data Fig. 7 [file 44318_2025_503_MOESM8_ESM.zip › Fig7H inclusion.tif]

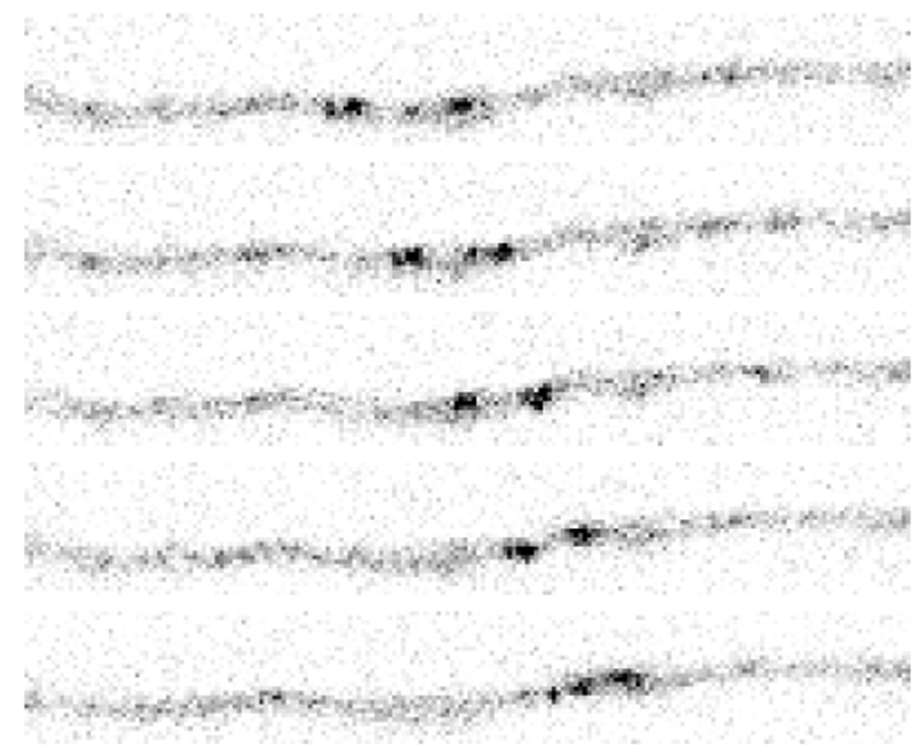

Supplement: Supplementary file 8 — Source data Fig. 7 [file 44318_2025_503_MOESM8_ESM.zip › Fig7H skipping.tif]

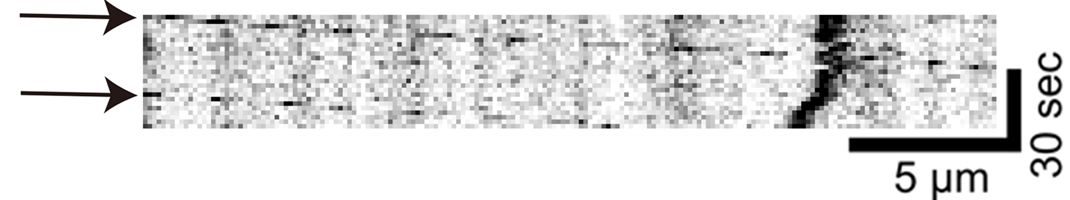

Supplement: Supplementary file 8 — Source data Fig. 7 [file 44318_2025_503_MOESM8_ESM.zip › Fig7I Inclusion.tif]

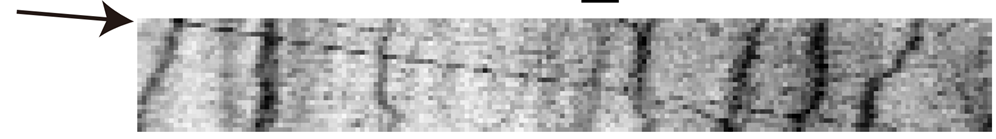

Supplement: Supplementary file 8 — Source data Fig. 7 [file 44318_2025_503_MOESM8_ESM.zip › Fig7I Skipping.tif]

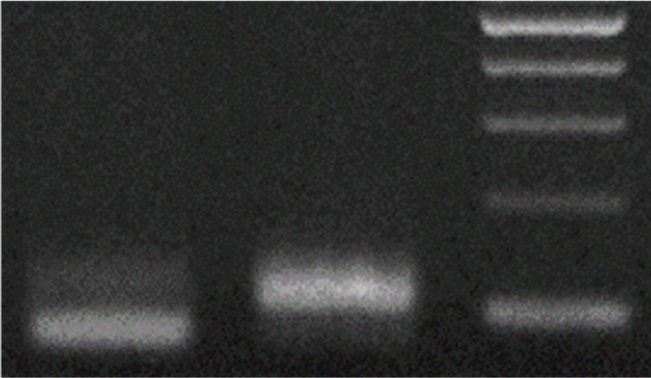

Supplement: Supplementary file 8 — Source data Fig. 7 [file 44318_2025_503_MOESM8_ESM.zip › Fig7K.tif]

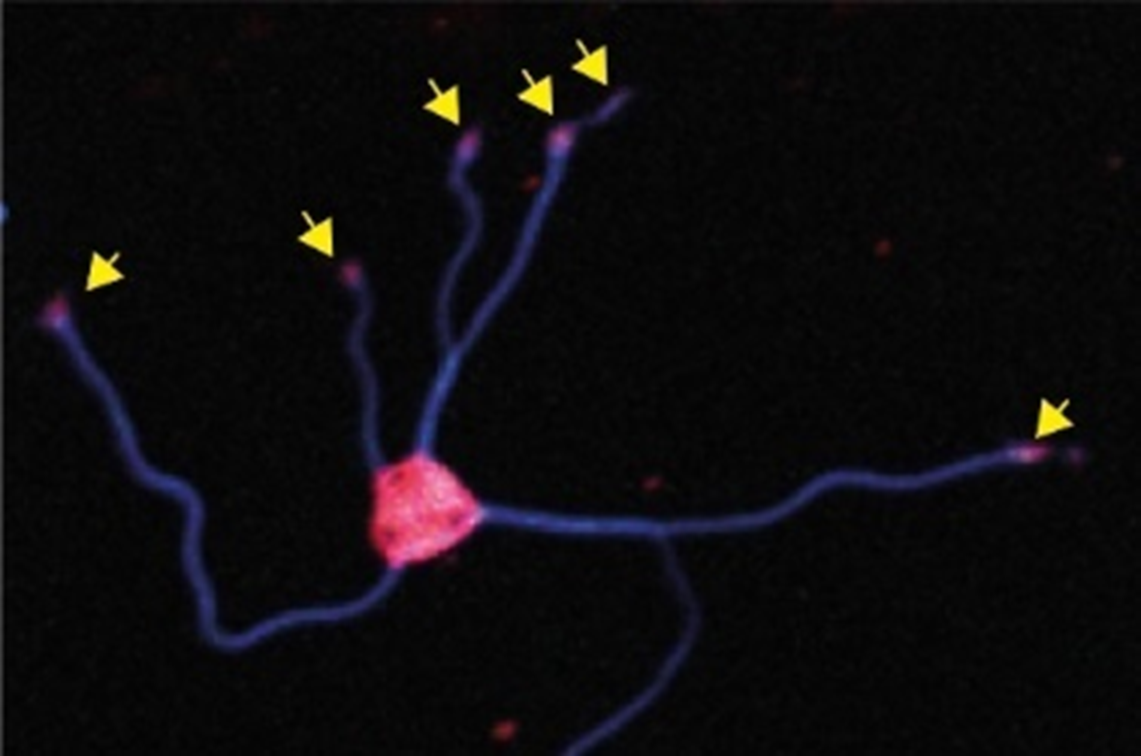

Supplement: Supplementary file 8 — Source data Fig. 7 [file 44318_2025_503_MOESM8_ESM.zip › Fig7M inclusion.tif]

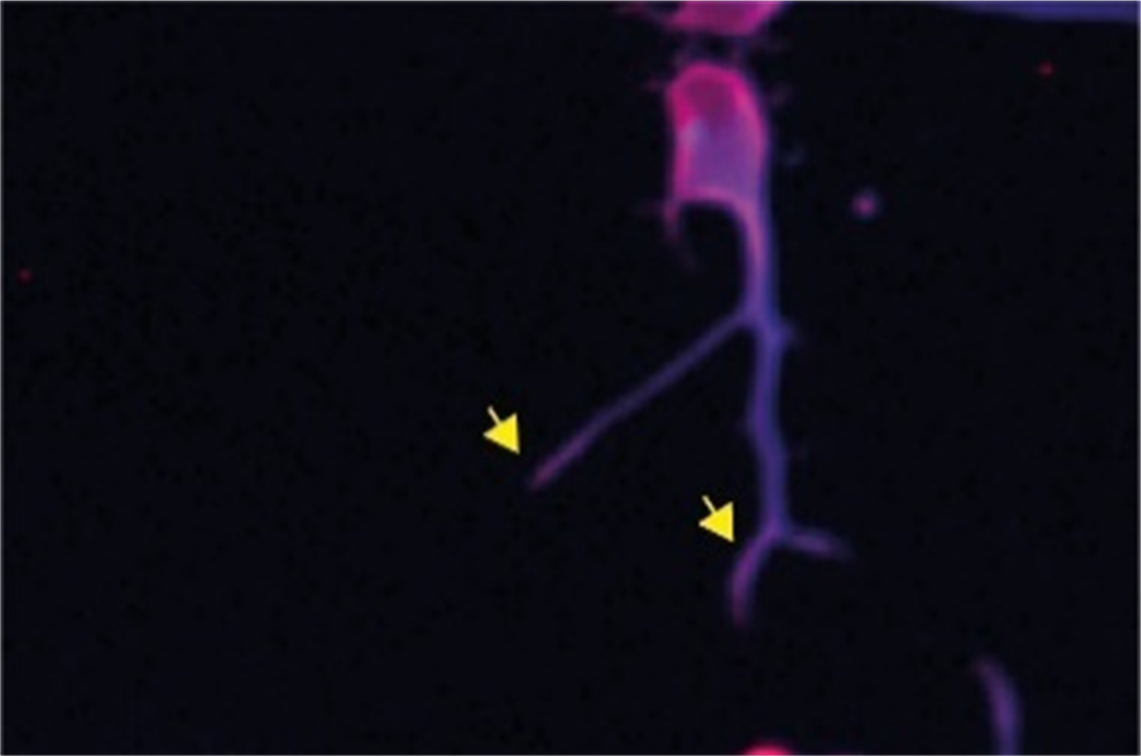

Supplement: Supplementary file 8 — Source data Fig. 7 [file 44318_2025_503_MOESM8_ESM.zip › Fig7M skipping.tif]

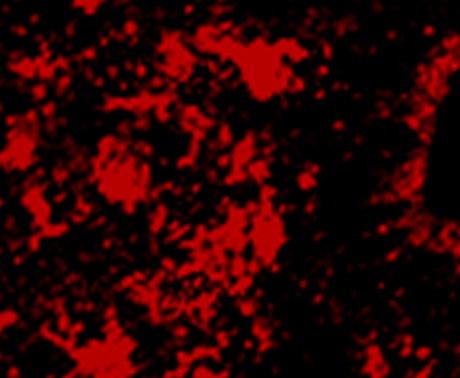

Supplement: Supplementary file 9 — Source data Fig. 8 [file 44318_2025_503_MOESM9_ESM.zip › Fig8A Ad lib Agrp.tif]

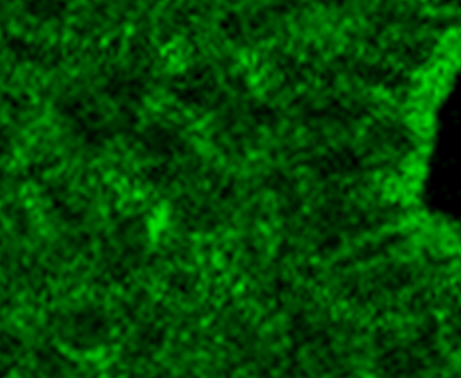

Supplement: Supplementary file 9 — Source data Fig. 8 [file 44318_2025_503_MOESM9_ESM.zip › Fig8A Ad lib Fto.tif]

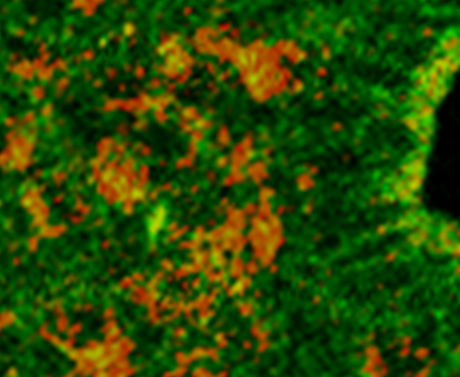

Supplement: Supplementary file 9 — Source data Fig. 8 [file 44318_2025_503_MOESM9_ESM.zip › Fig8A Ad lib merge.tif]

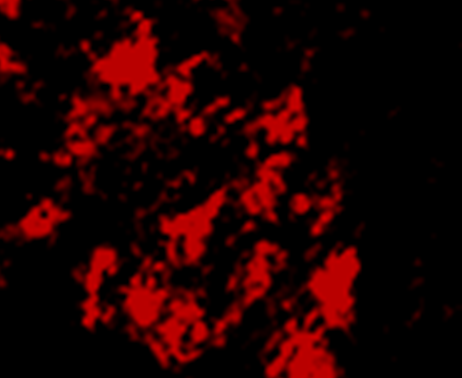

Supplement: Supplementary file 9 — Source data Fig. 8 [file 44318_2025_503_MOESM9_ESM.zip › Fig8A Fasting Agrp.tif]

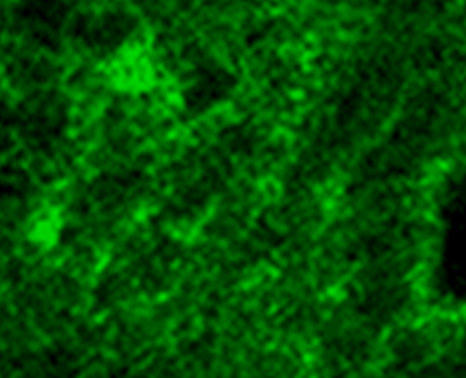

Supplement: Supplementary file 9 — Source data Fig. 8 [file 44318_2025_503_MOESM9_ESM.zip › Fig8A Fasting Fto.tif]

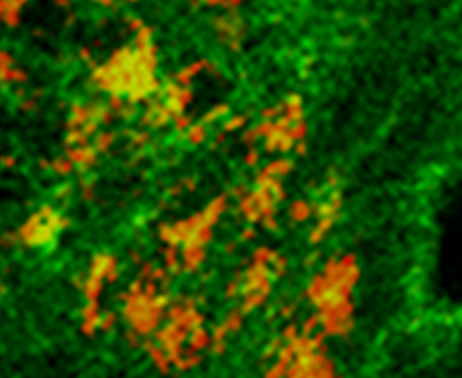

Supplement: Supplementary file 9 — Source data Fig. 8 [file 44318_2025_503_MOESM9_ESM.zip › Fig8A Fasting merge.tif]

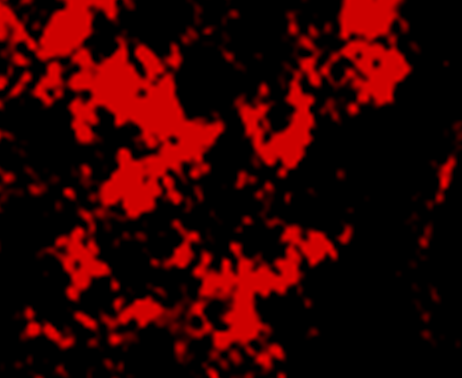

Supplement: Supplementary file 9 — Source data Fig. 8 [file 44318_2025_503_MOESM9_ESM.zip › Fig8A Refeeding Agrp.tif]

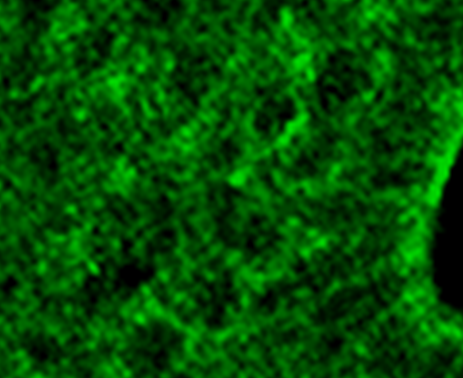

Supplement: Supplementary file 9 — Source data Fig. 8 [file 44318_2025_503_MOESM9_ESM.zip › Fig8A Refeeding Fto.tif]

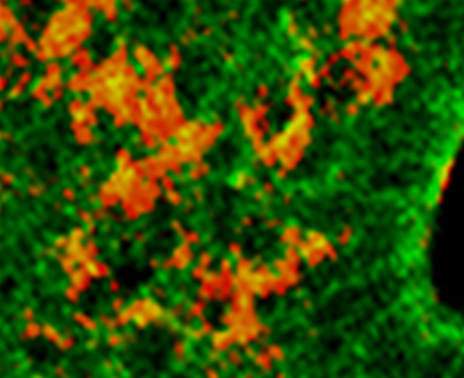

Supplement: Supplementary file 9 — Source data Fig. 8 [file 44318_2025_503_MOESM9_ESM.zip › Fig8A Refeeding merge.tif]

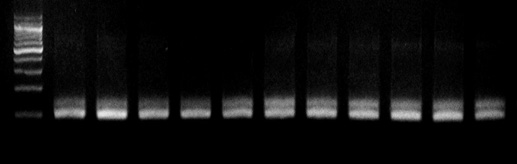

Supplement: Supplementary file 9 — Source data Fig. 8 [file 44318_2025_503_MOESM9_ESM.zip › Fig8C.jpg]

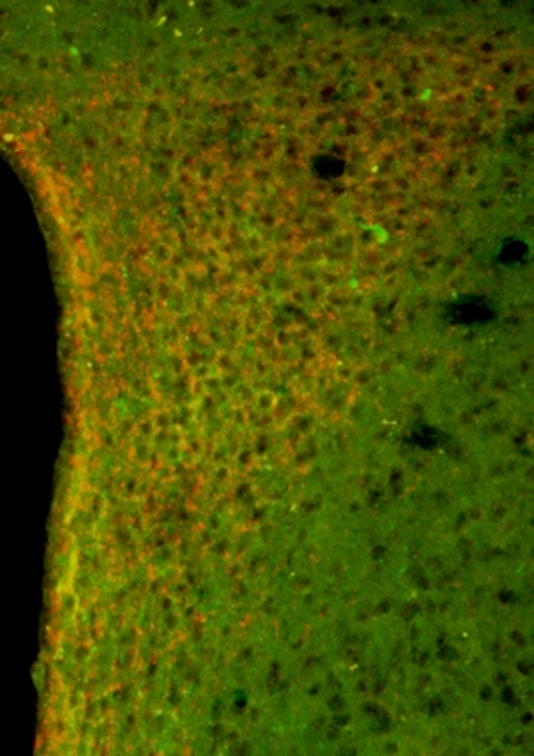

Supplement: Supplementary file 9 — Source data Fig. 8 [file 44318_2025_503_MOESM9_ESM.zip › Fig8F Ad lib merge.tif]

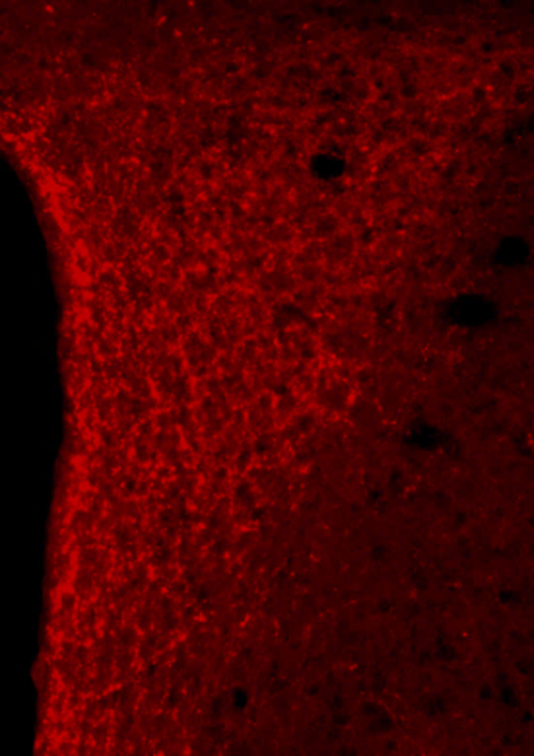

Supplement: Supplementary file 9 — Source data Fig. 8 [file 44318_2025_503_MOESM9_ESM.zip › Fig8F Ad lib NPY.tif]

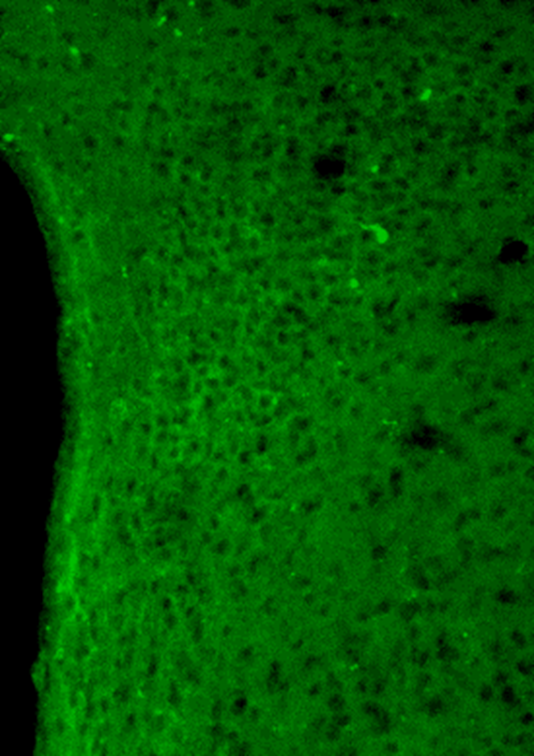

Supplement: Supplementary file 9 — Source data Fig. 8 [file 44318_2025_503_MOESM9_ESM.zip › Fig8F Ad lib Secretogranin II.tif]

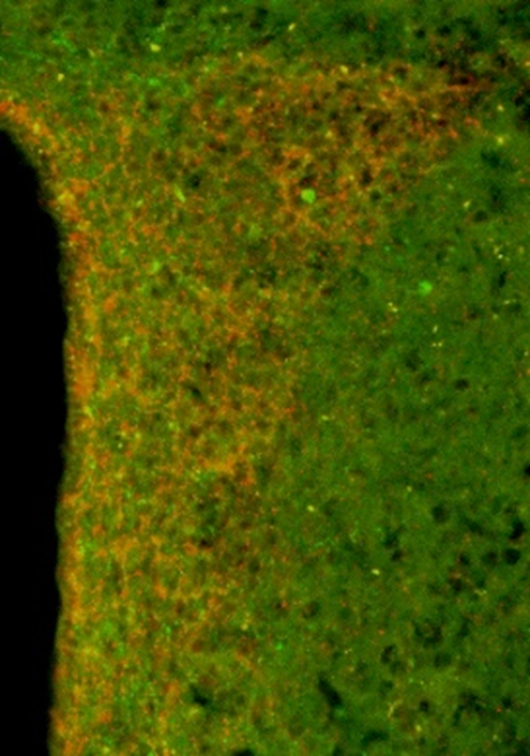

Supplement: Supplementary file 9 — Source data Fig. 8 [file 44318_2025_503_MOESM9_ESM.zip › Fig8F Fasting Merge.tif]

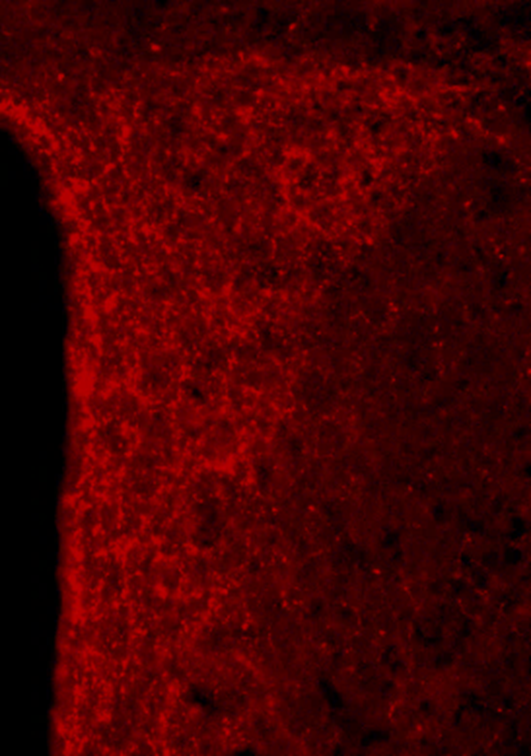

Supplement: Supplementary file 9 — Source data Fig. 8 [file 44318_2025_503_MOESM9_ESM.zip › Fig8F Fasting NPY.tif]

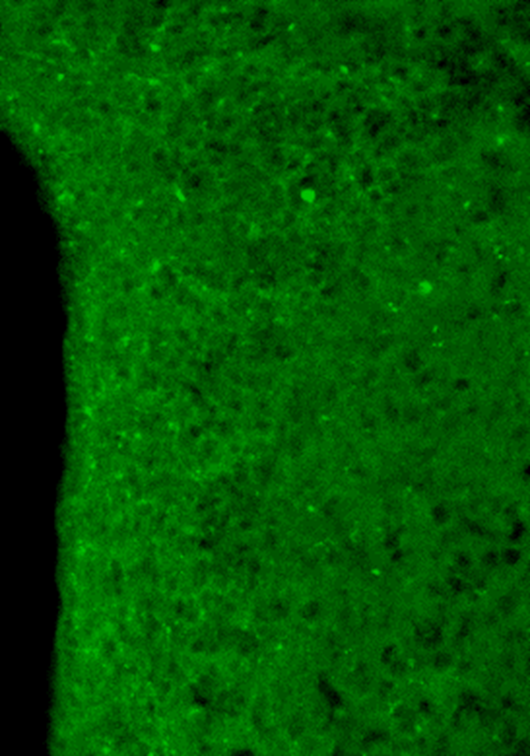

Supplement: Supplementary file 9 — Source data Fig. 8 [file 44318_2025_503_MOESM9_ESM.zip › Fig8F Fasting Secretogranin II.tif]

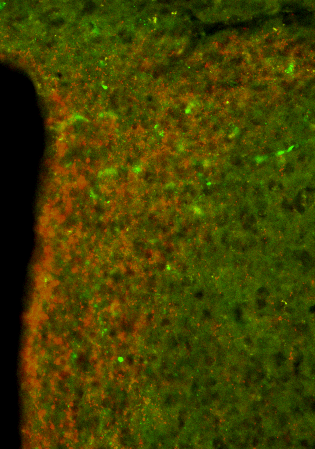

Supplement: Supplementary file 9 — Source data Fig. 8 [file 44318_2025_503_MOESM9_ESM.zip › Fig8F Refeeding merge.tif]

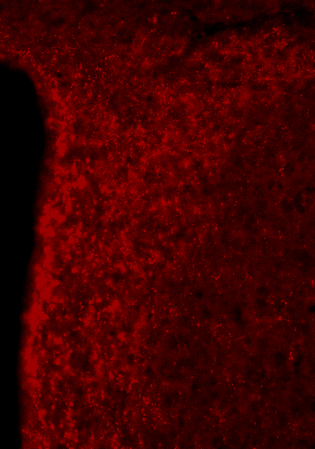

Supplement: Supplementary file 9 — Source data Fig. 8 [file 44318_2025_503_MOESM9_ESM.zip › Fig8F Refeeding NPY.tif]

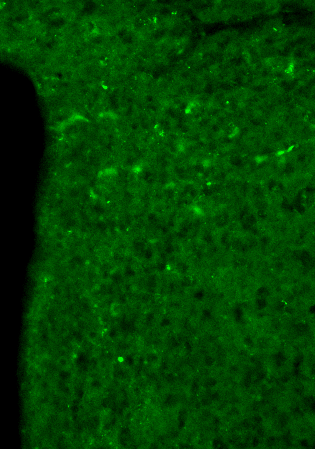

Supplement: Supplementary file 9 — Source data Fig. 8 [file 44318_2025_503_MOESM9_ESM.zip › Fig8F Refeeding Secretogranin II.tif]
