# Supplementary material for: FTO promotes weight gain via altering Kif1a splicing and axonal vesicle trafficking in AgRP neurons
Source: EMBO J. 2025 Jul 9;44(18):4919–61. doi: 10.1038/s44318-025-00503-3 (PMC12436618; doi:10.1038/s44318-025-00503-3)
Supplement: Supplementary file 10 — Expanded View Figures [file 44318_2025_503_MOESM10_ESM.pdf]

Expanded View Figures

Figure EV1. Distribution of FTO in the hypothalamic feeding center.

(**A**, **B**) In situ hybridization of *Fto*. *Fto* mRNA is abundantly localized in the arcuate nucleus (ARC), ventromedial hypothalamus (VMH) and paraventricular hypothalamus (PVH). Scale bar: (**A**) 500 µm, (**B**) 100 µm. (**C**) Transmitted light microscopy image of X-gal staining (light blue) in *Fto-LacZ* mice. *LacZ* expression patterns in *Fto-LacZ* mice recapitulated *Fto* expression patterns observed by *Fto* in situ hybridization. Scale bar: 500 µm. (**D–G**) Color-reversed transmitted light microscopy image of X-gal staining (red) and NPY-hrGFP fluorescence (green) (**D**) and immunofluorescence (green) of POMC (**E**), NUCB2 (**F**), or tyrosine hydroxylase (TH) (**G**) in the ARC of *Fto-lacZ* mice. Scale bar: 30 µm. (**H**) The percentage of X-gal-expressing neurons among neurons expressing NPY-hrGFP or immunoreactive for POMC, NUCB2, or TH in the ARC. $n = 3$ for each group; error bars represent SEM. (**I**) Confocal microscopy image of X-gal staining (blue) and NPY-hrGFP fluorescence (green) of *Fto-LacZ*/NPY-hrGFP mouse. Scale bar: 30 µm. 3V: third ventricle. (**J–O**) Color-reversed transmitted light microscopy image of X-gal staining (red) and the immunofluorescence (green) of oxytocin (**J**), vasopressin (**K**), NUCB2 (**L**), TH (**M**), corticotropin-releasing hormone (CRH) (N), or thyrotropin-releasing hormone (TRH) (**O**) in the PVH of *Fto-lacZ* mice. Scale bar: 30 µm. (**P**) The percentage of X-gal-expressing neurons among neurons immunoreactive for TRH ($n = 3$), TH ($n = 3$), oxytocin ($n = 3$), NUCB2 ($n = 4$), AVP ($n = 3$), or CRH ($n = 3$) in the PVH. Error bars represent SEM.

© The Author(s)

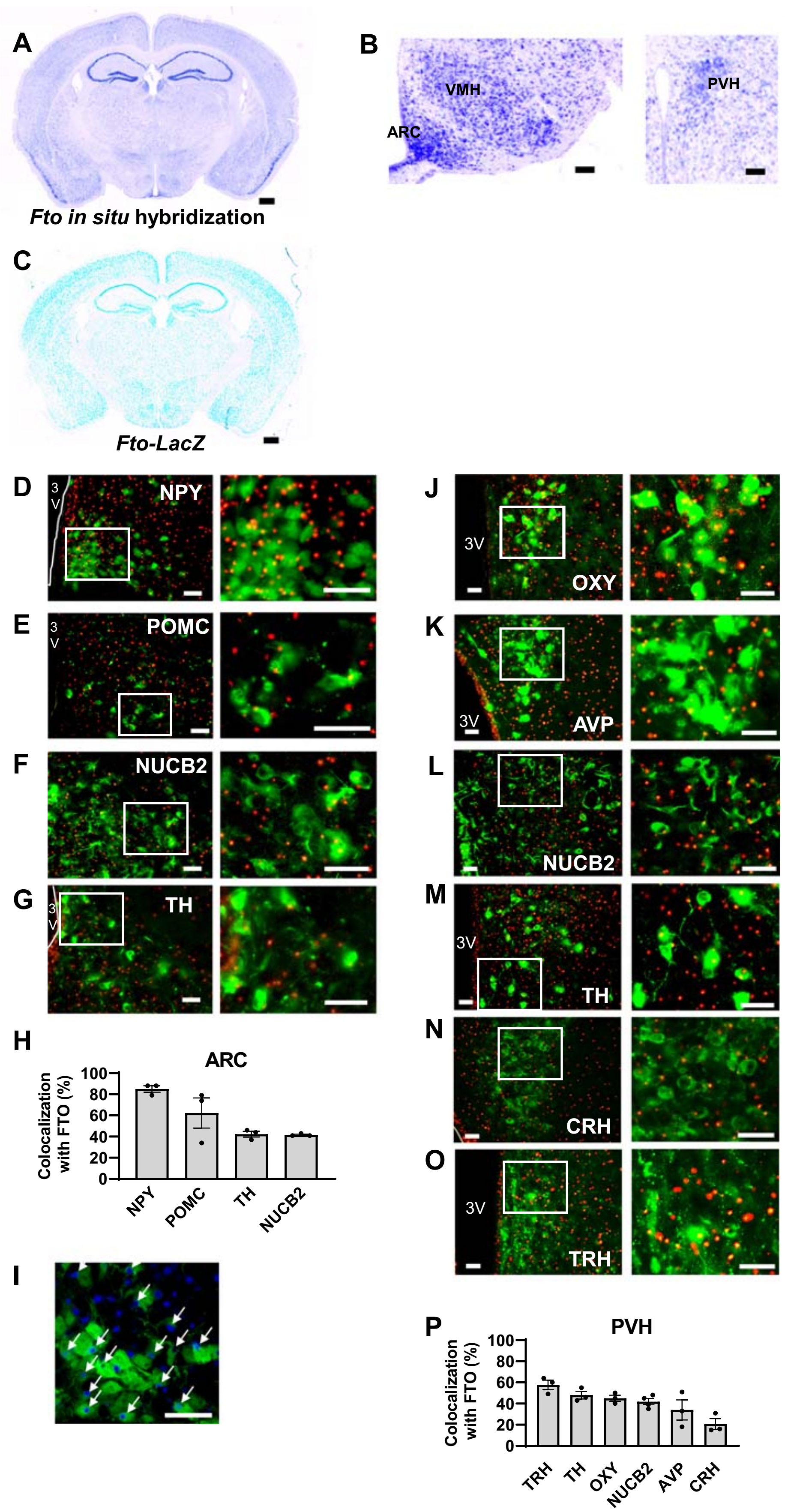
A
B
VMH
ARC
PVH
Fto in situ hybridization
C
Fto-LacZ
D
3V
NPY
E
POMC
F
NUCB2
G
TH
H
ARC
Colocalization with FTO (%)
100
80
60
40
20
0
NPY
POMC
TH
NUCB2
I
J
OXY
K
AVP
L
NUCB2
M
TH
N
CRH
O
TRH
P
PVH
TRH
TH
OXY
NUCB2
AVP
CRH

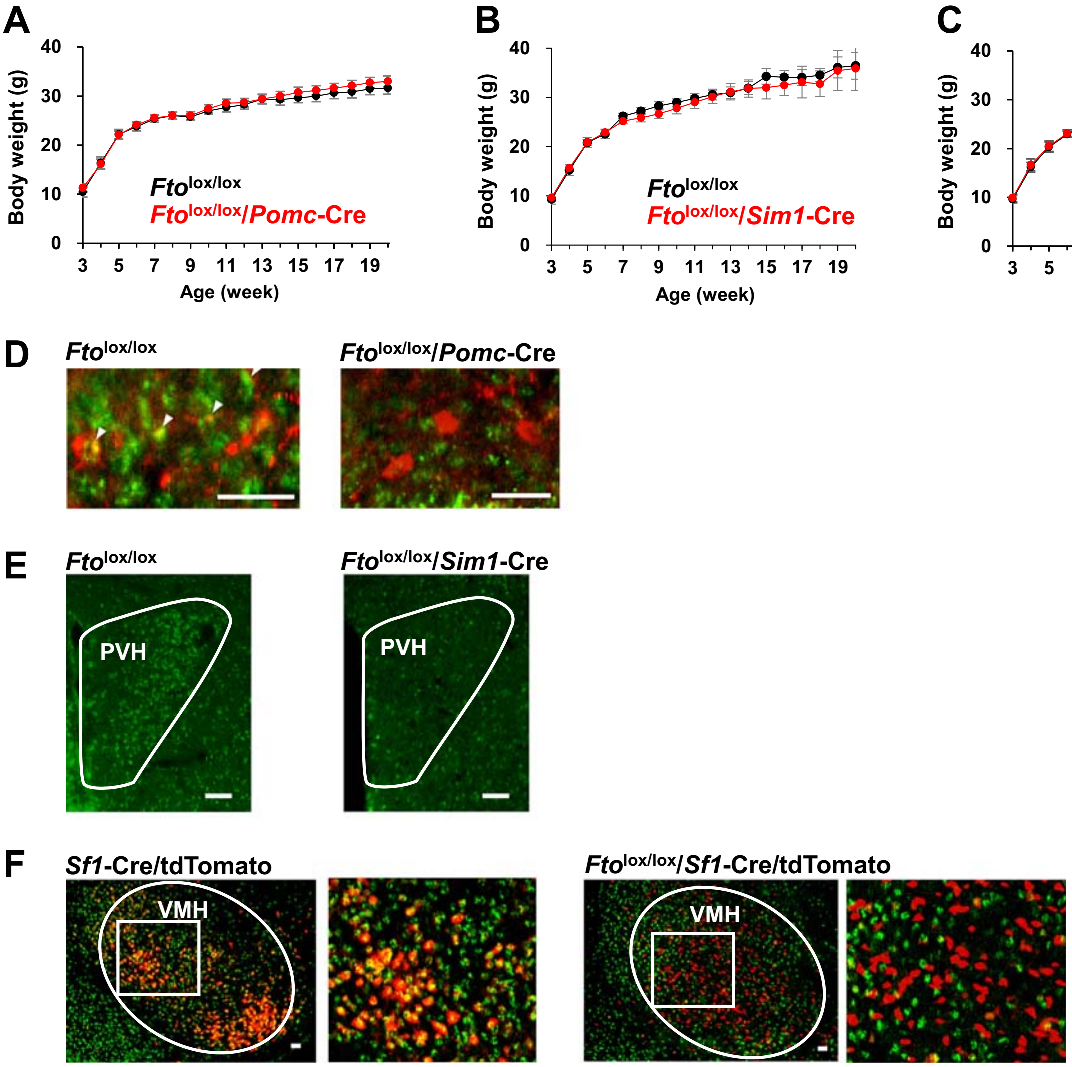

Figure EV2. ***Fto* conditional knockout mice specific for hypothalamic neurons.**

(**A–C**) The body weights of the male mice lacking *Fto* specifically in *Pomc*-Cre ($n = 10$–12) (*Fto*$^{lox/lox}$/*Pomc*-Cre) (**A**), *Sim1*-Cre ($n = 3$–13) (*Fto*$^{lox/lox}$/*Sim1*-Cre) (**B**), and *Sf1*-Cre (*Fto*$^{lox/lox}$/*Sf1*-Cre) ($n = 6$–13) (**C**) were comparable to those of control (*Fto*$^{lox/lox}$) mice. Error bars represent SEM. Data were analyzed using unpaired Welch's *t* test. (**D–F**) FTO immunofluorescence (green) was colocalized with POMC-immunofluorescence (red) (arrow) in *Fto*$^{lox/lox}$ mice but not in *Fto*$^{lox/lox}$/*Pomc*-Cre mice (**D**). FTO immunofluorescence in the PVH was abundant and sparse in *Fto*$^{lox/lox}$ mouse and *Fto*$^{lox/lox}$ /*Sim1*-Cre mouse, respectively (**E**). FTO immunofluorescence colocalized with tdTomato fluorescence in the VMH of *Sf1*-Cre/tdTomato mice but not in VMH of *Fto*$^{lox/lox}$/*Sf1*-Cre/tdTomato mice (**F**). Scale bar: 30 μm.

© The Author(s)

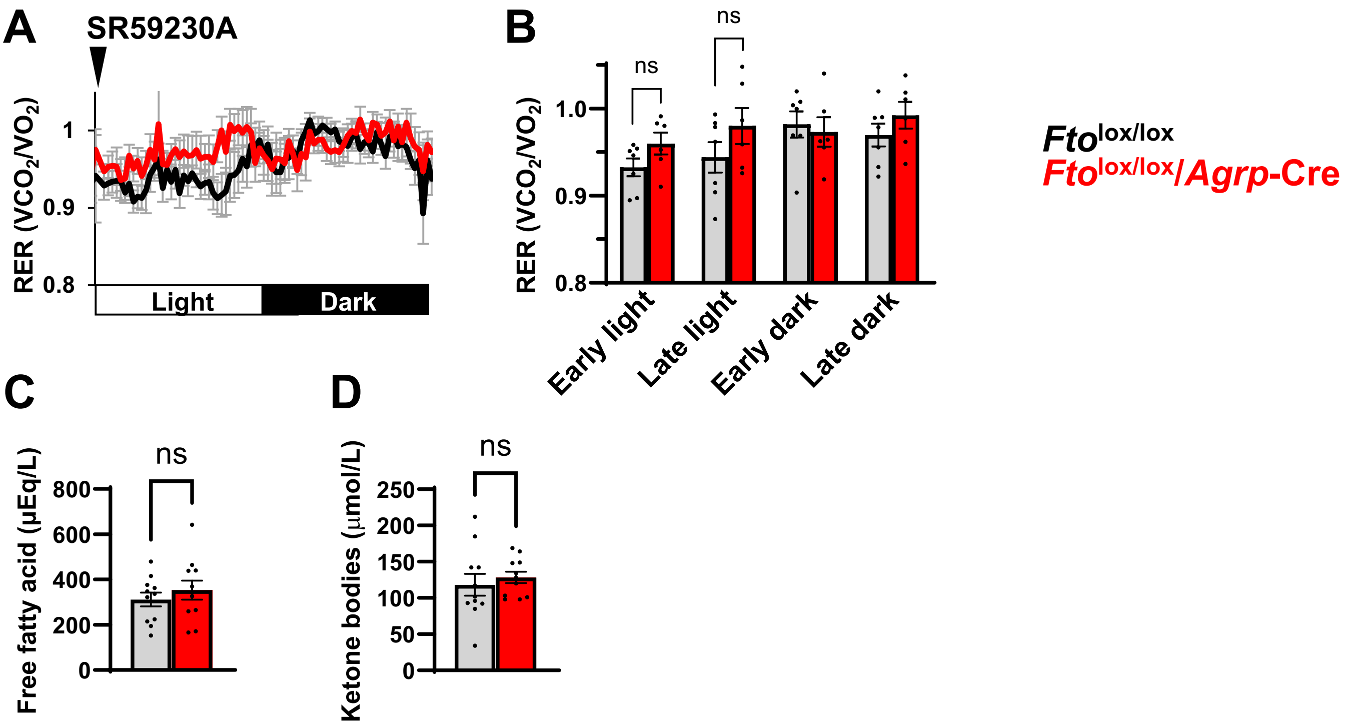

Figure EV3. Administration of a β3 adrenergic receptor antagonist affected the levels of respiratory exchange ratio (RER), free fatty acid, and ketone bodies in *Fto*$^{lox/lox}$/*Agrp*-Cre mice.

(**A**) The RERs of *Fto*$^{lox/lox}$ ($n = 7$, black) and *Fto*$^{lox/lox}$/*Agrp*-Cre ($n = 6$, red) male mice injected intraperitoneally with SR59230A, a β3 adrenergic receptor antagonist (10 mg/kg body weight), at ZT0 (arrowhead). Error bars represent SEM. Data were analyzed using unpaired Student's *t* test. (**B**) Average RER of *Fto*$^{lox/lox}$ ($n = 7$, gray) and *Fto*$^{lox/lox}$/*Agrp*-Cre ($n = 6$, red) male mice injected with SR59230A at ZT0. Error bars represent SEM. Data were analyzed using unpaired Student's *t* test. (**C**, **D**) Levels of free fatty acids (**C**) and ketone bodies (**D**) in the serum of male *Fto*$^{lox/lox}$ (gray, $n = 11$) and *Fto*$^{lox/lox}$/*Agrp*-Cre (red, $n = 11$) mice injected with SR59230A at ZT0 and with blood collected at ZT2. Error bars represent SEM. Data were analyzed using unpaired Student's *t* test.

 © The Author(s)

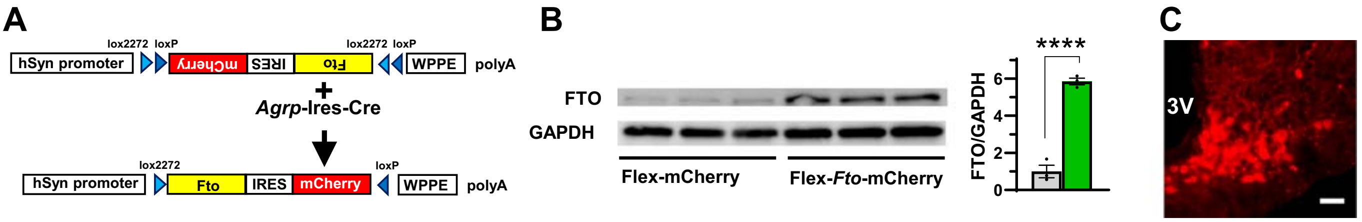

Figure EV4. Generation of AgRP neuron-specific *Fto* overexpression mice.

Diagram of the Cre-inducible *Fto* overexpression AAV vector, AAV-hSyn-Flex-*Fto*-mCherry (**A**). Validation of the specific overexpression of FTO was conducted by western blotting using ARC samples from *Agrp*-Ires-Cre mice injected with either Flex-mCherry (control) or Flex-*Fto*-mCherry 4 weeks earlier. FTO protein levels were significantly higher in *Agrp*-Ires-Cre mice injected with AAV-hSyn-Flex-*Fto*-mCherry (green, $n = 3$) compared to those injected with AAV-hSyn-Flex-mCherry (gray, $n = 3$) (**B**). Error bars represent SEM. Data were analyzed using unpaired Student's t test; ****$P < 0.001$ (exact P value: 0.0002). Immunohistochemistry of mCherry using *Agrp*-Ires-Cre mice injected with AAV-hSyn-Flex-*Fto*-mCherry (**C**). Scale bar: 30 μm.

© The Author(s)

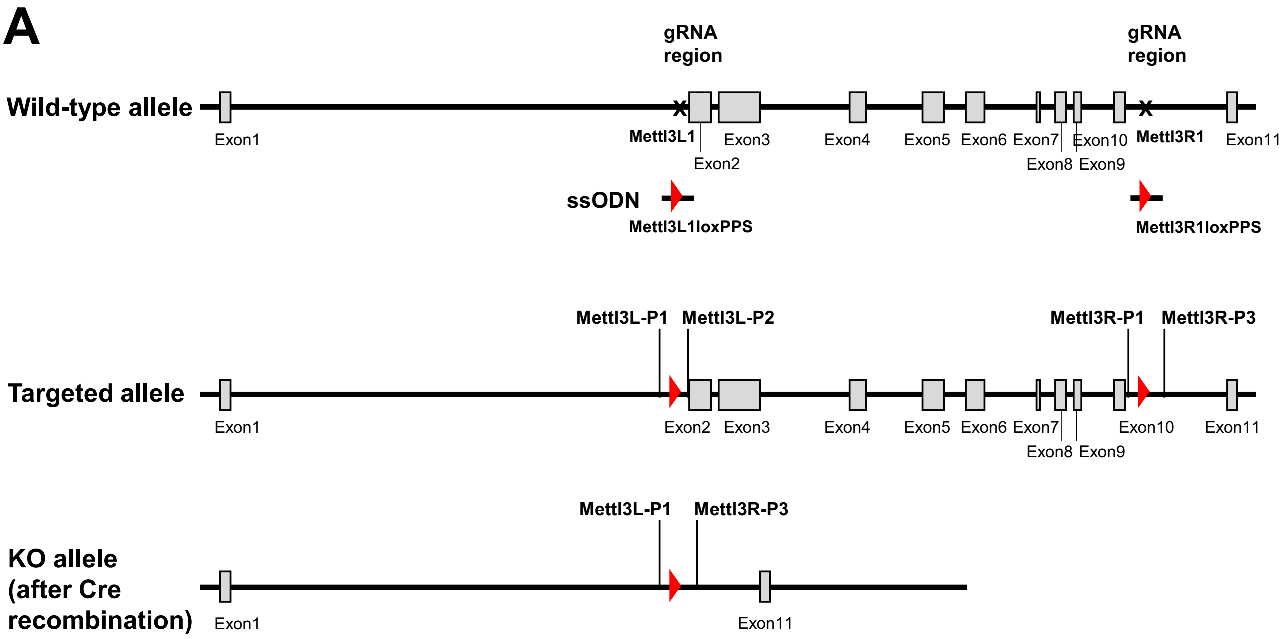

B

Locus	Target	ssODN
Left loxP	**Mettl3L1**<br>5'-AGTGCTGCCATGTGAATGAA-3'	**Mettl3L1loxPPS**<br>5'-GT*A*G*TTCTCTGGAAAATCCAGGATTATT TTGGCAAAACAGCAAGTGCTGCCATGTGAAT GGGATCCATAACTTCGTATAATGTATGCTATA CGAAGTTATAAAGGTGATCTAGAGCTAACGC TGGTCAGAGACCCTGCTTGAAGTGAAAGATG TGTG*T*G*C-3'
Right loxP	**Mettl3R1**<br>5'-ATACATTCTGGTAGTCTAGA-3'	**Mettl3R1loxPPS**<br>5'-CA*A*A*AAACCCTTTATGGACAAAGAC AGATGTCACTGAGTTGTATACATTCTGGTAGT CTAATAACTTCGTATAATGTATGCTATACGAA GTTATGTCGACGATGGCTTAAGCTTTACCAGA ATCTACAACATTCATTAGAACCCAAAGGGCTG TTCT*T*A*A-3'

Astarisks indicate a phosphothioate bond.

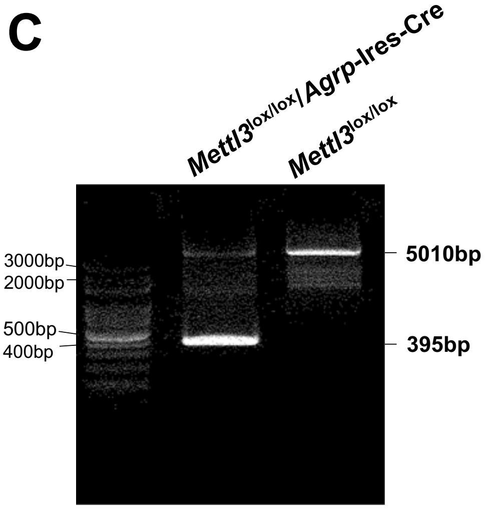

Figure EV5. AgRP neuron-specific *Mettl3* knockout mice did not show body weight phenotype.

(**A**) Schematic illustration of generation of a conditional allele at the *Mettl3* locus. Two loxP sites were inserted into *Mettl3* intron 1 and intron 10. (**B**) Sequences of ssODNs with 5′- and 3′-homology arms flanking loxP and a restriction site. Asterisks indicate phosphorothioate bonds. (**C**) Genomic DNA was extracted from the ARC, and PCR was performed using the Mettl3L-P1 and Mettl3R-P3 primers. A deletion-specific DNA fragment (395 bp) was detected in *Mettl3*$^{lox/lox}$/*Agrp*-Ires-Cre mouse samples, whereas a non-deletion-specific DNA fragment (5010 bp) was detected in *Mettl3*$^{lox/lox}$ mouse samples.

 © The Author(s)

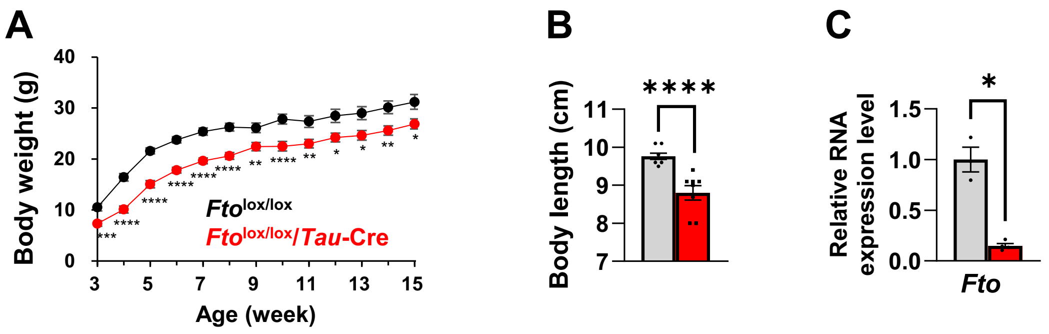

Figure EV6. *Tau*-Cre specific *Fto*-knockout mice had reduced body weight and body length.

Body weight ($n = 9$– 19) (**A**) and body length ($n = 8$) (**B**) of 10-week-old of *Fto*$^{lox/lox}$/*Tau*-Cre mice were significantly lower than those of control (*Fto*$^{lox/lox}$) mice. *Fto* mRNA expression levels in the ARC of *Fto*$^{lox/lox}$/*Tau*-Cre mice ($n = 4$) were significantly lower than in the ARC of *Fto*$^{lox/lox}$ mice ($n = 3$) (**C**). Error bars represent SEM. Data were analyzed using unpaired Student's *t* test (**A**) and unpaired Welch's *t* test (**B**, **C**); *$P < 0.05$, **$P < 0.01$, ***$P < 0.005$, ****$P < 0.001$ (exact *P* values: 0.003 [3-week-old], 6.18×10^{-7} [4-week-old], 1.77×10^{-8} [5-week-old], 3.37×10^{-8} [6-week-old], 6.97×10^{-7} [7-week-old], 8.37×10^{-6} [8-week-old], 0.008 [9-week-old], 0.0007 [10-week-old], 0.006 [11-week-old], 0.013 [12-week-old], 0.011 [13-week-old], 0.008 [14-week-old], 0.022 [15-week-old]) (**A**); 0.0009 (**B**); 0.017 (**C**)).

© The Author(s)

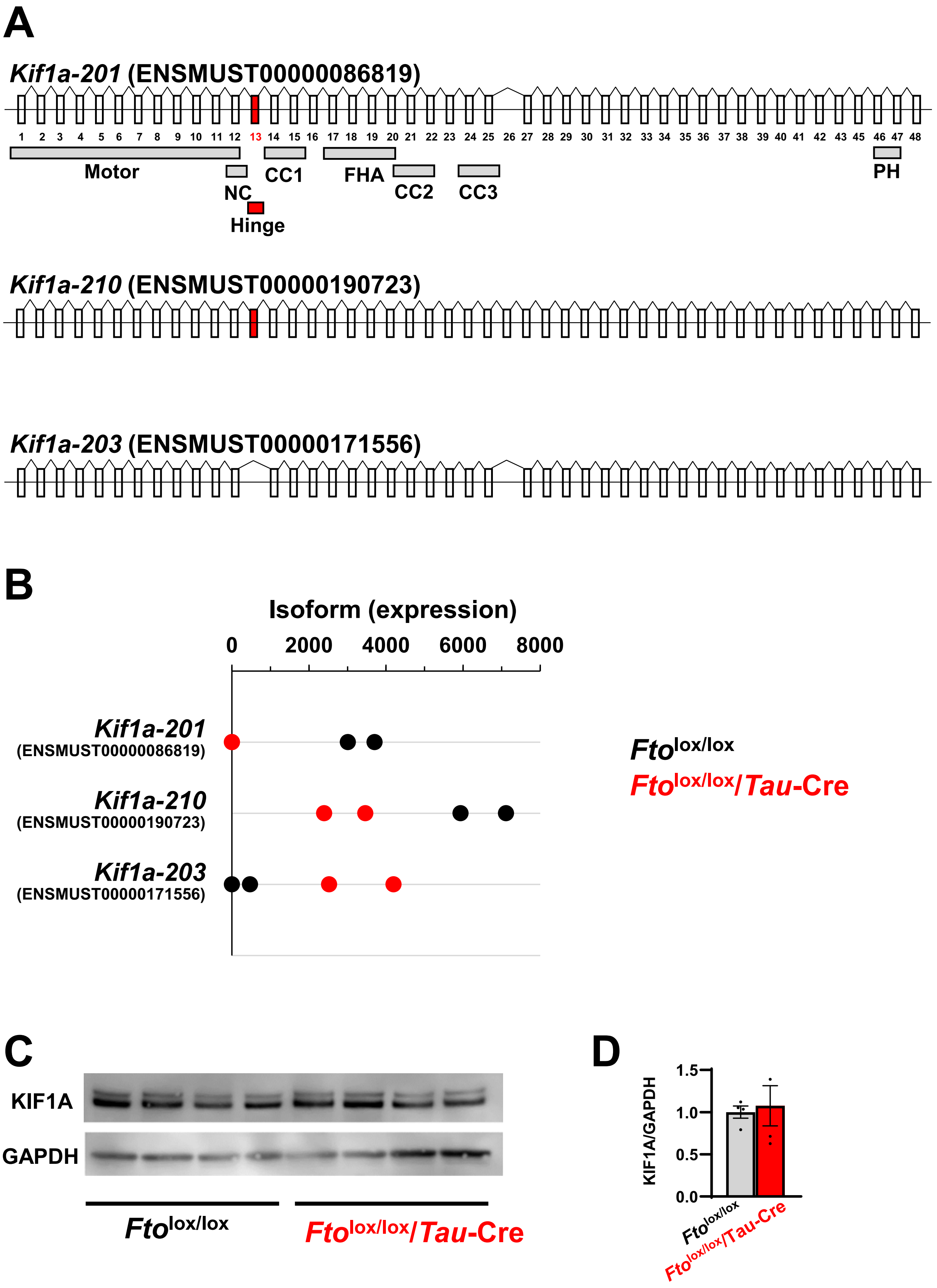
A
Kif1a-201 (ENSMUST00000086819)
1 2 3 4 5 6 7 8 9 10 11 12 13 14 15 16 17 18 19 20 21 22 23 24 25 26 27 28 29 30 31 32 33 34 35 36 37 38 39 40 41 42 43 45 46 47 48
Motor
NC
Hinge
CC1
FHA
CC2
CC3
PH
Kif1a-210 (ENSMUST00000190723)
Kif1a-203 (ENSMUST00000171556)
B
Isoform (expression)
0 2000 4000 6000 8000
Kif1a-201 (ENSMUST00000086819)
Kif1a-210 (ENSMUST00000190723)
Kif1a-203 (ENSMUST00000171556)
Fto lox/lox
Fto lox/lox/Tau-Cre
C
KIF1A
GAPDH
Fto lox/lox
Fto lox/lox/Tau-Cre
D
KIF1A/GAPDH
1.5
1.0
0.5
0.0
Fto lox/lox
Fto lox/lox/Tau-Cre

 © The Author(s)

Figure EV7. Lack of FTO altered alternative splicing but not protein expression of *Kif1a*.

(**A**) Alternative splicing generates cDNAs of *Kif1a* splice variants, *Kif1a-201*, *Kif1a-210*, and *Kif1a-203*. (**B**) Expression levels of each splice variant in the mediobasal hypothalamus of *Fto*$^{lox/lox}$ (black) and *Fto*$^{lox/lox}$/*Tau*-Cre (red) mice. Two pooled samples were analyzed for each genotype. (**C**, **D**) Western blotting of KIF1A in the mediobasal hypothalamus of *Fto*$^{lox/lox}$ and *Fto*$^{lox/lox}$/*Tau*-Cre mice (**C**). There was no significant difference in the KIF1A protein levels between *Fto*$^{lox/lox}$ ($n = 4$) and *Fto*$^{lox/lox}$/*Tau*-Cre ($n = 4$) mice (**D**). Error bars represent SEM. Data were analyzed using unpaired Student's t test.

© The Author(s)

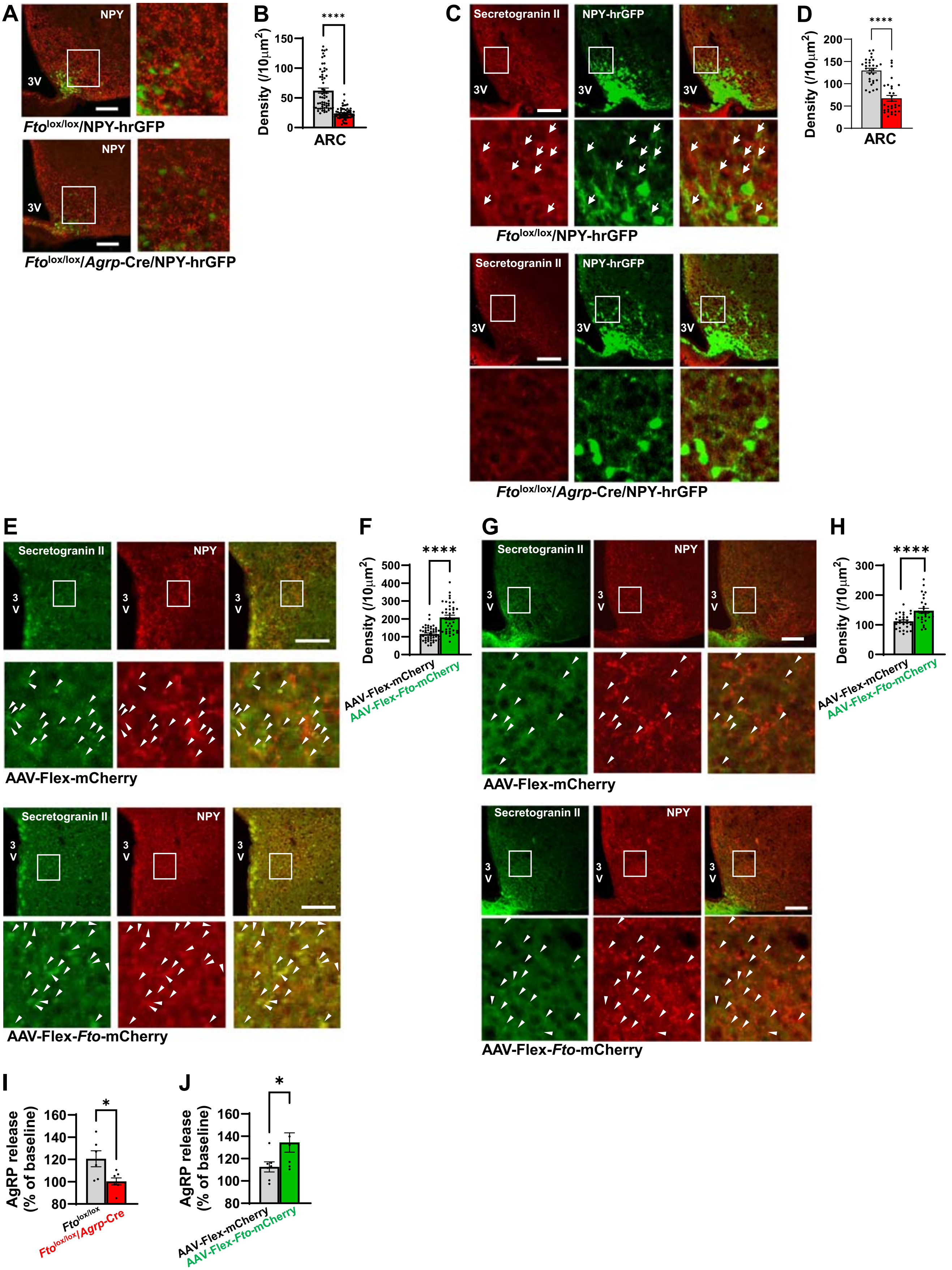
A
NPY
3V
Fto
Ftolox/lox/NPY-hrGFP
Ftolox/lox/Agrp-Cre/NPY-hrGFP
B
Density (/10μm2)
ARC
C
Secretogranin II
NPY-hrGFP
D
E
F
AAV-Flex-mCherry
AAV-Flex-Fto-mCherry
G
H
I
AgRP release (% of baseline)
Ftolox/lox
Ftolox/lox/Agrp-Cre
J

 © The Author(s)

Figure EV8. FTO is indispensable for the axonal transport of dense-core vesicles (DCV) containing NPY and AgRP.

(**A**) NPY immunofluorescence (red) and the cell bodies of NPY/AgRP neurons visualized using NPY-hrGFP (green). (**B**) Density of NPY immunofluorescence in the ARC of *Fto*$^{lox/lox}$/NPY-hrGFP (gray, $n = 60$) and *Fto*$^{lox/lox}$/*Agrp*-Cre/NPY-hrGFP mice (red, $n = 60$). Error bars represent SEM. Data were analyzed using unpaired Welch's *t* test; ****$P < 0.001$ (exact *P* value: 2.72×10^{-12}). (**C**, **D**) Immunofluorescence of secretogranin II (red) and NPY-hrGFP fluorescence (green) in the ARC (**C**). Density of secretogranin II immunofluorescence in the ARC of *Fto*$^{lox/lox}$/NPY-hrGFP (gray, $n = 30$) and *Fto*$^{lox/lox}$/*Agrp*-Cre/NPY-hrGFP (red, $n = 30$) mice (**D**). Error bars represent SEM. Data were analyzed using unpaired Student's *t* test; ****$P < 0.001$ (exact *P* value: 4.83×10^{-10}). (**E–H**) Immunofluorescence of secretogranin II (green) and NPY (red) in the PVH (**E**) and ARC (**G**) of *Agrp*-Ires-Cre mice injected with AAV-hSyn-Flex-mCherry or AAV-hSyn-Flex-*Fto*-mCherry. Density of secretogranin II immunofluorescence in the PVH (**F**, $n = 48$ sites from three mice (AAV-hSyn-flex-mCherry) and 39 sites from three mice (AAV-hSyn-Flex-*Fto*-mCherry)) and ARC (**G**, $n = 28$ sites from three mice per AAV) of *Agrp*-Ires-Cre mice injected with AAV-hSyn-Flex-mCherry or AAV-Flex-*Fto*-mCherry. Error bars represent SEM. Data were analyzed using unpaired Welch's *t* test; ****$P < 0.001$ (exact *P* values: 1.56×10^{-8} (**F**), 0.0006 (**H**)). (**I**, **J**) AgRP release from brain slices of *Fto*$^{lox/lox}$ ($n = 6$) and *Fto*$^{lox/lox}$/*Agrp*-Cre/NPY-hrGFP ($n = 7$) mice (**I**) and of *Agrp*-Ires-Cre mice injected with AAV-hSyn-Flex-mCherry (gray, $n = 7$) or AAV-hSyn-Flex-*Fto*-mCherry (green, $n = 7$) (**J**) in response to a solution containing a low concentration (2.5 mM) of glucose and 100 μM glutamate. Scale bar: 100 μm. Error bars represent SEM. Data were analyzed using unpaired Student's *t* test (**J**); *$P < 0.05$ (exact *P* values: 0.019 (**I**), 0.044 (**J**)).

© The Author(s)

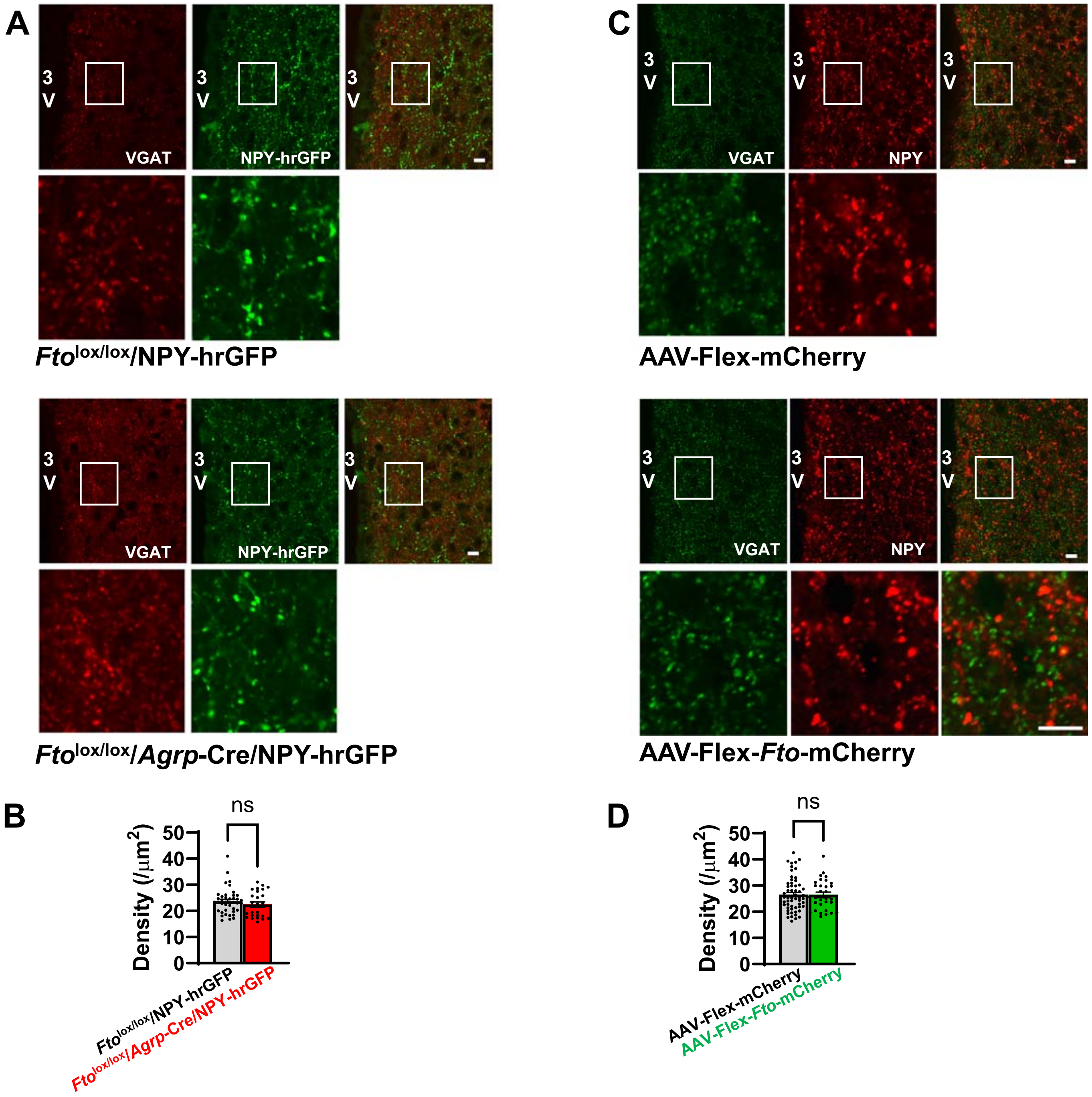

Figure EV9. FTO does not alter vesicular GABA transporter (VGAT) density in NPY/AgRP fibers in the PVH.

(**A**) VGAT immunofluorescence (red) and NPY-hrGFP (green) in the PVH of *Fto*$^{lox/lox}$/NPY-hrGFP and *Fto*$^{lox/lox}$/*Agrp*-Cre/NPY-hrGFP mice. Scale bar: 10 μm. (**B**) Density of VGAT immunofluorescence in the PVH adjacent to the NPY fibers of *Fto*$^{lox/lox}$/NPY-hrGFP (gray, $n = 3$ mice, 40 areas) and *Fto*$^{lox/lox}$/*Agrp*-Cre/NPY-hrGFP (red, $n = 3$ mice, 26 areas) mice. Error bars represent SEM. Data were analyzed using unpaired Student's *t* test. (**C**) VGAT immunofluorescence (green) and NPY immunofluorescence (red) in the PVH of *Agrp*-Ires-Cre mice injected with AAV-hSyn-Flex-mCherry and AAV-hSyn-Flex-*Fto*-mCherry. Scale bar: 10 μm. (**D**) Density of VGAT immunofluorescence in the PVH adjacent to NPY fibers in the PVH of *Agrp*-Ires-Cre mice injected with AAV-hSyn-Flex-mCherry (gray, $n = 3$ mice, 59 sites) or AAV-hSyn-Flex-*Fto*-mCherry (green, $n = 3$ mice, 30 sites). Error bars represent SEM. Data were analyzed using unpaired Student's *t* test.

 © The Author(s)

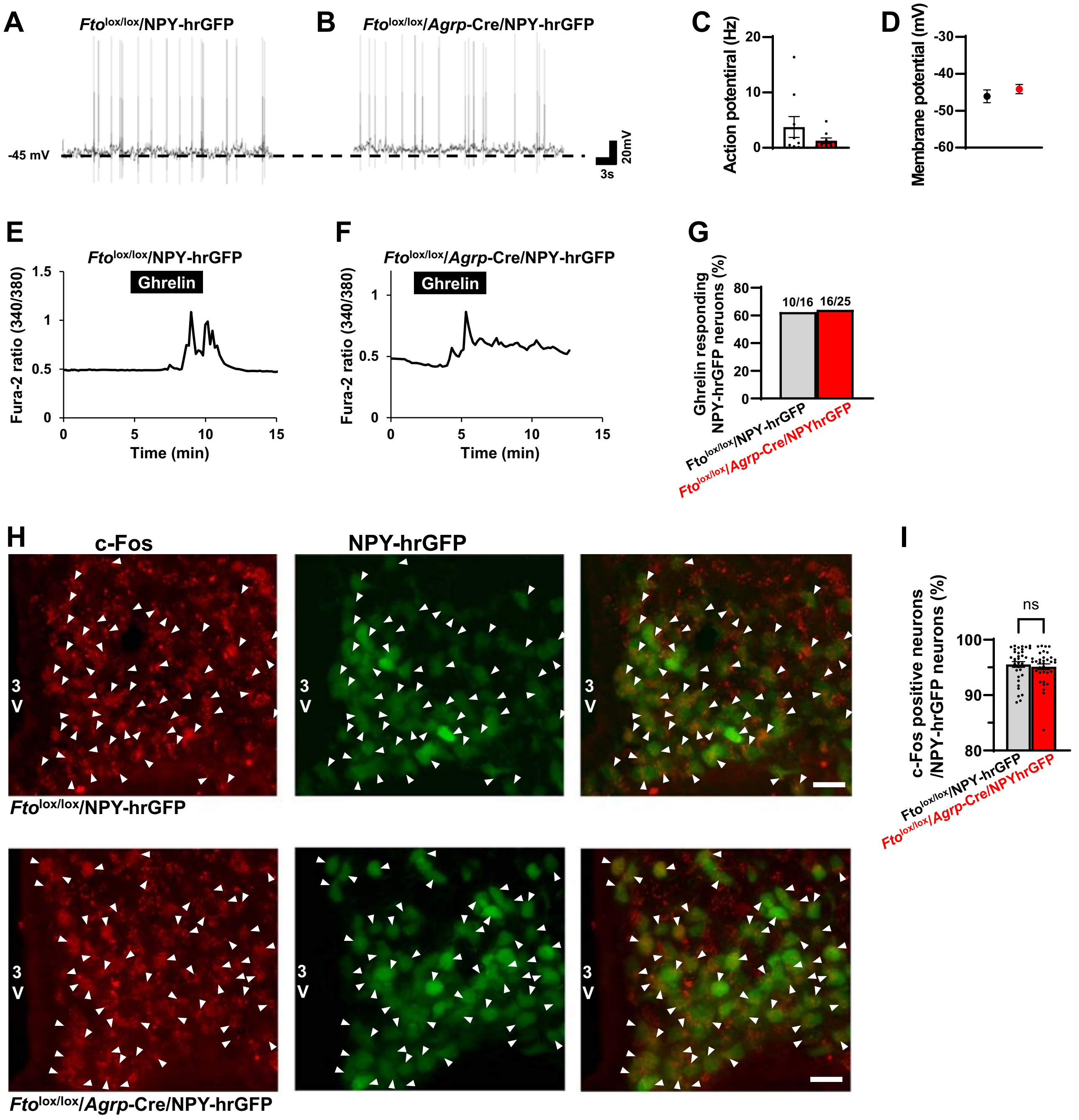

Figure EV10. Cellular activity of NPY/AgRP neurons lacking FTO is normal.

(**A–D**) Whole-cell patch-clamp recordings of AgRP neurons identified by NPY-hrGFP fluorescence from *Fto*$^{lox/lox}$/NPY-hrGFP (**A**) and *Fto*$^{lox/lox}$/*AgRP*-Cre/NPY-hrGFP mice (**B**). There were no significant differences in the action potential (**C**) or membrane potential (**D**) between *Fto*$^{lox/lox}$/NPY-hrGFP ($n = 9$) and *Fto*$^{lox/lox}$/*AgRP*-Cre/NPY-hrGFP (red) mice ($n = 9$). Error bars represent SEM. (**E–G**) Fura-2 calcium imaging of isolated NPY-hrGFP neurons was performed. Representative Fura-2 ratio traces of NPY-hrGFP neurons from *Fto*$^{lox/lox}$/NPY-hrGFP (**E**) and *Fto*$^{lox/lox}$/*AgRP*-Cre/NPY-hrGFP mice subjected to ghrelin at 10^{-10} M. (**G**) The percentage of NPY-hrGFP neurons responded to ghrelin. The number above each bar indicates the number of NPY-hrGFP neurons responded to ghrelin over the number of NPY-hrGFP neurons analyzed. (**H, I**) c-Fos-immunofluorescence (red) in NPY-hrGFP (green) neurons of overnight-fasted *Fto*$^{lox/lox}$/NPY-hrGFP and *Fto*$^{lox/lox}$/*AgRP*-Cre-NPY-hrGFP mice (**H**). Arrowheads indicate neurons exhibiting both c-Fos and NPY-hrGFP. Scale bar. 20 µm. The percentage of c-Fos-positive neurons among NPY-hrGFP neurons in *Fto*$^{lox/lox}$/NPY-hrGFP ($n = 36$ unilateral sections from three mice) and *Fto*$^{lox/lox}$/*AgRP*-Cre-NPY-hrGFP ($n = 35$ unilateral sections from three mice) mice (**I**). Error bars represent SEM. Data were analyzed using unpaired Student's *t* test; exact *P* value: 0.566.

© The Author(s)

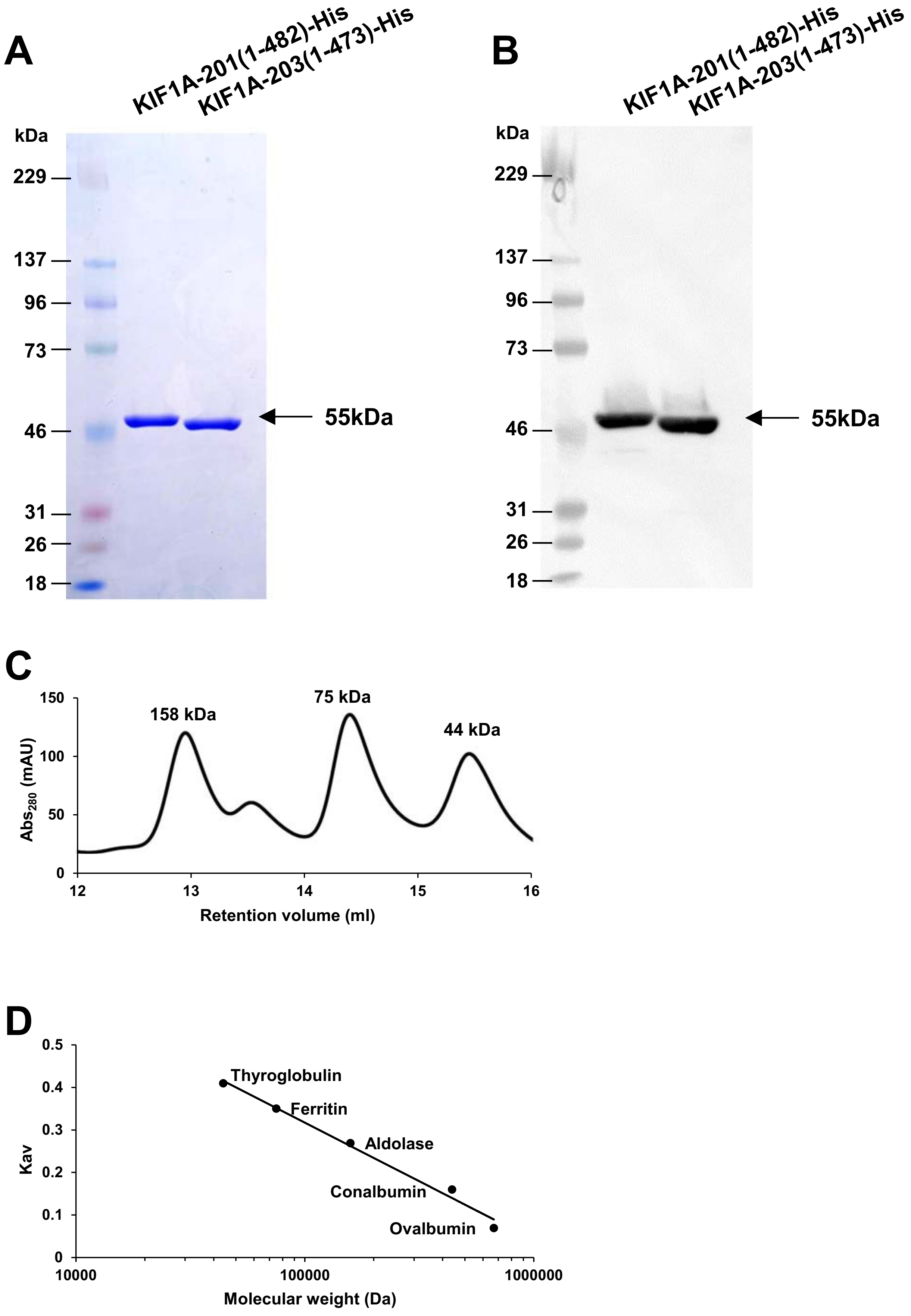
A
KIF1A-201(1-482)-His
KIF1A-203(1-473)-His
kDa
229
137
96
73
46
31
26
18
55kDa
B
KIF1A-201(1-482)-His
KIF1A-203(1-473)-His
kDa
229
137
96
73
46
31
26
18
55kDa
C
158 kDa
75 kDa
44 kDa
Abs280 (mAU)
150
100
50
0
12
13
14
15
16
Retention volume (ml)
D
Thyroglobulin
Ferritin
Aldolase
Conalbumin
Ovalbumin
Kav
0.5
0.4
0.3
0.2
0.1
0
10000
100000
1000000
Molecular weight (Da)

© The Author(s)

Figure EV11. Validation of recombinant KIF1A proteins, and molecular weight determination by size-exclusion chromatography.

(**A**, **B**) Validation of recombinant KIF1A proteins. Recombinant KIF1A-201(1-482)-His and KIF1A-203(1-473)-His proteins (2.7 µg each) were analyzed by SDS-PAGE. The gel was stained with Coomassie Brilliant Blue R-250 (**A**). Following SDS-PAGE, proteins were transferred onto a membrane for western blotting using anti-KIF1A antibody (**B**). A protein molecular weight marker image, captured under white light exposure, was overlaid on the western blotting image. These analyses confirmed that the KIF1A proteins were highly purified. (**C**, **D**) Molecular weight determination by size-exclusion chromatography. Retention volume of standard globular proteins in size-exclusion chromatography (**C**). The calibration curve was generated by plotting the Kav value for each standard against its corresponding molecular weight, and used to determine the molecular weight of the KIF1A fragments (**D**).

© The Author(s)

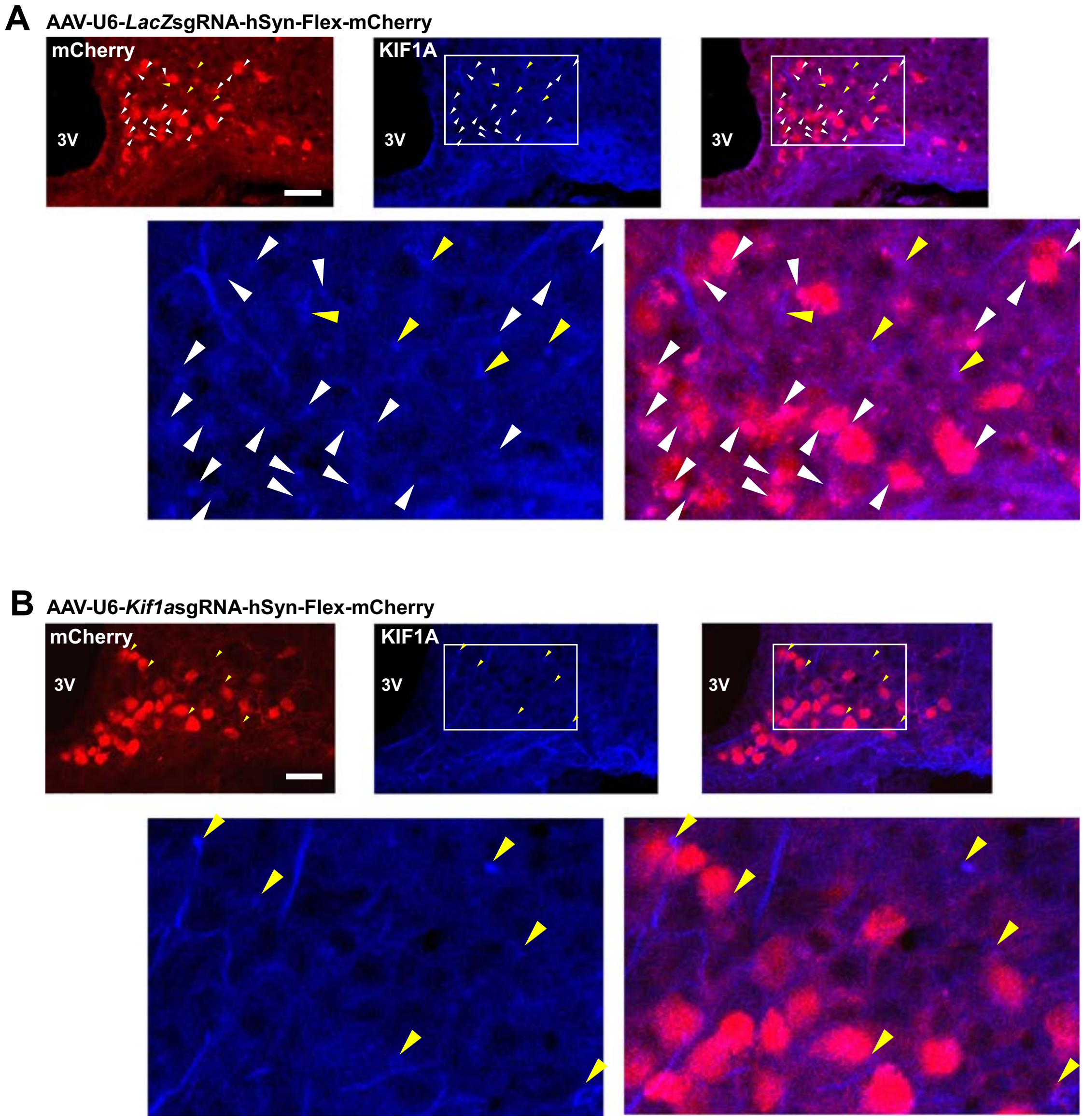

Figure EV12. Validation of KIF1A knockdown in AgRP neuron-specific *Kif1a* knockdown mouse.

AAV-U6-*Kif1a*sgRNA-hSyn-Flex-mCherry (**A**) or AAV-U6-LacZsgRNA-hSyn-Flex-mCherry (**B**) was injected into the ARC of Rosa26-LSL-Cas9 knock-in/*Agrp*-Ires-Cre mice. The mCherry expression (red), KIF1A immunofluorescence (blue), and merged images are shown. White arrowheads indicate neurons exhibiting both types of fluorescence, while yellow arrowheads indicate neurons exhibiting KIF1A fluorescence only. Scale bar: 30 μm.

 © The Author(s)

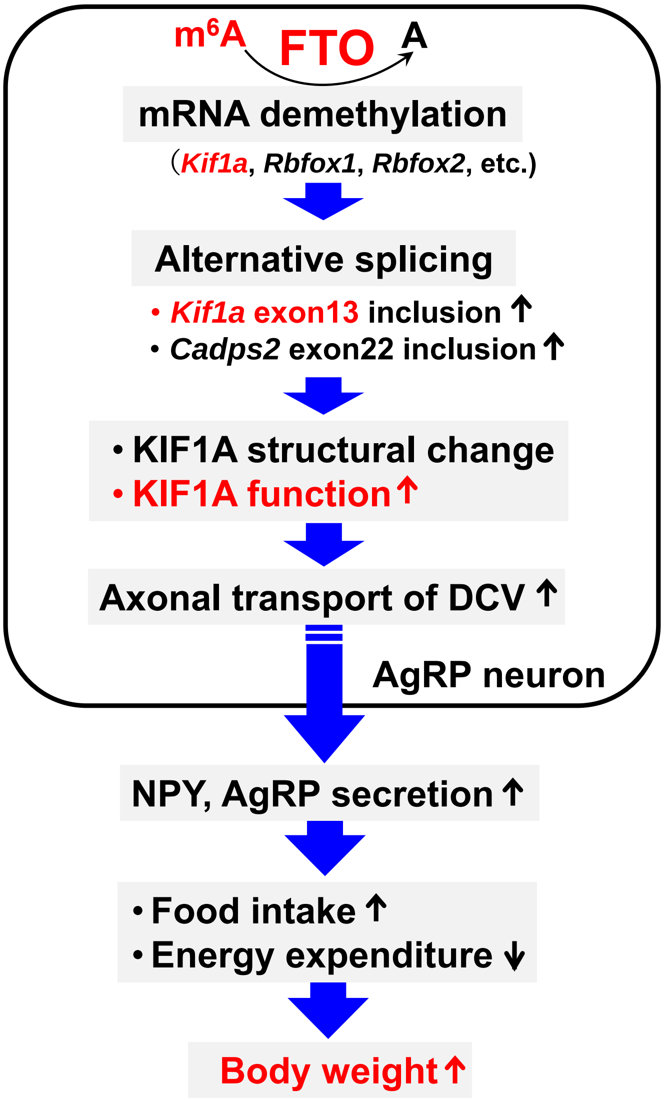

Figure EV13. Schematic representation of the role of FTO in AgRP neurons.

FTO in AgRP neurons controls body weight. In AgRP neurons, FTO demethylates mRNAs associated with membrane trafficking and alternative splicing, including *Kif1a*, *Rbfox1*, and *Rbfox2*. Then, alternative splicing of *Kif1a* exon 13 inclusion and *Cadps2* exon 22 inclusion is upregulated. *Kif1a* exon 13 inclusion alters the protein structure of KIF1A and reinforces KIF1A function. As a result, FTO enhances the axonal transport of DCVs and the secretion of NPY and AgRP, thereby increasing food intake, decreasing energy expenditure, and increasing body weight.

© The Author(s)
